# MAP kinases associate with high molecular weight multiprotein complexes

**DOI:** 10.1093/jxb/erx424

**Published:** 2017-12-12

**Authors:** Carlton J Bequette, Sarah R Hind, Sarah Pulliam, Rebecca Higgins, Johannes W Stratmann

**Affiliations:** Department of Biological Sciences, University of South Carolina, Columbia, USA

**Keywords:** Arabidopsis, MAP kinase signaling, MAPK, MAPKK, multiprotein complex, phosphorylation, scaffold, signal transduction, size-exclusion chromatography, tomato

## Abstract

Plant responses to the environment and developmental processes are mediated by a complex signaling network. The *Arabidopsis thaliana* mitogen-activated protein kinases (MAPKs) MPK3 and MPK6 and their orthologs in other plants are shared signal transducers that respond to many developmental and environmental signals and thus represent highly connected hubs in the cellular signaling network. In animals, specific MAPK signaling complexes are assembled which enable input-specific protein–protein interactions and thus specific signaling outcomes. In plants, not much is known about such signaling complexes. Here, we report that MPK3, MPK6, and MPK10 orthologs in tomato, tobacco, and Arabidopsis as well as tomato MAPK kinase 4 (MKK4) associate with high molecular weight (~250–550 kDa) multiprotein complexes. Elicitation by the defense-associated peptides flg22 and systemin resulted in phosphorylation and activation of the monomeric MAPKs, whereas the complex-associated MAPKs remained unphosphorylated and inactive. In contrast, treatment of tomato cells with a phosphatase inhibitor resulted in association of phosphorylated MPK1/2 with the complex. These results demonstrate that plant MAPKs and MAPKKs dynamically assemble into stable multiprotein complexes and this may depend on their phosphorylation status. Identification of the constituents of these multiprotein complexes promises a deeper understanding of signaling dynamics.

## Teaser

Depending on their phosphorylation status, MAP kinases (MAPKs) dynamically associate with stable high molecular weight multiprotein complexes.

## Introduction

Some mitogen-activated protein kinases (MAPKs) respond to almost any disturbance of cellular homoeostasis, ranging from the seemingly innocuous touch stimulus to severe abiotic and biotic stress factors such as ultraviolet light, extreme temperatures, pathogen infection, and herbivory (Ichimura *et al.*, 2000; Stratmann, 2008; Rodriguez *et al.*, 2010; Sinha *et al.*, 2011; Meng and Zhang, 2013; Bigeard *et al.*, 2015; Hettenhausen *et al.*, 2015; Xu and Zhang, 2015). MAPKs that are activated by stress and danger signals relay those signals through phosphorylation of either transcription factors, which regulate stress response genes, or extranuclear proteins involved in hormone biosynthesis and other cellular processes (Meng and Zhang, 2013). Two prominent MAPKs are MPK3 and MPK6 from *Arabidopsis thaliana* and their orthologs in other plants. In addition to regulating responses to environmental factors, these MAPKs are also involved in the regulation of developmental processes (Lampard *et al.*, 2008; Wang *et al.*, 2008; Zhou *et al.*, 2009; Beck *et al.*, 2010; Xu and Zhang, 2015). This astonishing pleiotropy is tied to signaling mechanisms that faithfully activate input-specific responses. Several mechanisms of MAPK signaling fidelity are known from animal systems, but they are poorly understood in plants.

MAPKs are components of the three-tiered MAPK module. MAPK kinase kinases (MAPKKKs) are activated by receptors and sensor proteins, and in turn activate MAPK kinases (MAPKKs) via phosphorylation. MAPKKs then phosphorylate and activate MAPKs. The number of putative MAPKKKs is large, with 60–80 in Arabidopsis (MAPK Group, 2002; Champion *et al.*, 2004), 75 in rice (Rao *et al.*, 2010), and 89 in tomato (Wu *et al.*, 2014), whereas the number of MAPKKs is confined to 10 in Arabidopsis, 8 in rice, 11 in poplar (MAPK Group, 2002; Hamel *et al.*, 2006), and only 5 in tomato (Wu *et al.*, 2014). In contrast, the number of MAPKs is generally higher than the number of MAPKKs, with 20 members in Arabidopsis, 15 in rice, 21 in poplar (MAPK Group, 2002; Hamel *et al.*, 2006), and 16 in tomato (Stulemeijer *et al.*, 2007; Kong *et al.*, 2012). Predictions strictly based on numbers seem to indicate that MAPKKs may function as convergence points for MAPKKKs, but the number of thoroughly studied MAPKKKs is too low to draw firm conclusions. Some MAPKs also represent convergence points for a number of MAPKKs. Arabidopsis MPK3 can interact with six and MPK6 with eight of the 10 MAPKKs (Andreasson and Ellis, 2010). The theoretical number of MAPK modules as a consequence of potential permutations of the three constituents is very high. Therefore, it is predicted that the same MAPK may engage with different substrate proteins depending on the specific MAPKKK/MAPKK module it associates with. Indeed, a number of different *in vivo* substrates of MPK3 and MPK6 were identified (Meng and Zhang, 2013) and hundreds of putative MPK3/6 substrates were found in *in vitro* proteomic analyses (Popescu *et al.*, 2009; Hoehenwarter *et al.*, 2013; Lassowskat *et al.*, 2014). In addition, MPK6 interacts with at least eight, and MPK3 with at least seven MAPK phosphatases that can dephosphorylate and inactivate them (Andreasson and Ellis, 2010; Bartels *et al.*, 2010; Umbrasaite *et al.*, 2010).

A fundamental question in signal transduction is how a signal relay such as a MAPK, which is shared among diverse input signals, can generate specific output responses. The critical factor appears to be signaling dynamics, namely how MAPKs dynamically interact with other proteins in a spatiotemporal manner. A number of hypotheses exist to explain how MAPKs can accomplish signaling fidelity. The above-mentioned combinatorial diversity of module constituents can potentially result in the assembly of a large number of alternative MAPK modules with differential functions. These modules may combine with additional proteins such as scaffold proteins or MAPK-inactivating phosphatases to form larger multiprotein complexes. Assembly of distinct modules and complexes may result in different MAPK activation kinetics (speed of activation, and amplitude and duration of activity) with consequences for the output responses. Mechanisms that interpret and process MAPK kinetics include interactions of MAPKs with different substrates and other proteins. These interactions may occur in various subcellular compartments and tissues. In addition, MAPKs and/or their interacting partners may undergo subcellular translocations.

In non-plant systems, scaffold proteins play an important role in organizing specific MAPK modules to achieve signaling specificity. A MAPK scaffold protein is generally a non-enzymatic protein that interacts with at least two components of a MAPK module and often also with additional regulatory proteins. In some cases scaffold proteins themselves have catalytic activity; for example, PBS2 from yeast functions as a scaffold and as a MAPK kinase (Posas and Saito, 1997). A scaffold protein may regulate the subcellular localization of MAPKs, participate in positive and negative feedback loops, alter accessibility of MAPKs for MAPK-regulating proteins such as phosphatases or MAPKKs, and insulate specific MAPK modules from interactions with other MAPK modules (Whitmarsh, 2006; Shaw and Filbert, 2009; Zeke *et al.*, 2009). Through these functions, a scaffold protein may have a profound influence on the outcome of MAPK signaling. The animal scaffold protein KSR binds all three components of the MAPK cascade and additional proteins such as 14-3-3 proteins, a phosphatase, and some heat shock proteins in a tissue-specific manner. In the absence of stimuli, it is present in the cytosol, but can translocate to the plasma membrane upon stimulation. Also, binding of its protein clients can occur in an input-specific manner (Kolch, 2005; Shaw and Filbert, 2009). These features highlight the dynamics of complex assembly and disassembly that are critical to achieve signaling fidelity. Recently, RACK1 was discovered as the first MAPK scaffold protein in plants (Cheng *et al.*, 2015; Su *et al.*, 2015). Arabidopsis RACK1 binds to the MAPKKK MEKK1, the MAPKKs MKK4 and MKK5, and the MAPKs MPK6 and MPK3.

We used gel filtration (GF) (size-exclusion chromatography) to separate high molecular weight (HMW) protein complexes from monomeric proteins in plant extracts. Probing GF fractions with antibodies against MAPKs, we identified various MAPKs as well as a MAPKK in HMW GF fractions (~250–550 kDa), indicating that MAPKs associate with multiprotein complexes. The presence of MAPK-containing multiprotein complexes was confirmed in five plant species from two different families (Solanaceae and Brassicaceae), showing that this is a general phenomenon. Only non-phosphorylated (i.e. inactive) MAPKs are present in the HMW GF fractions, unless dephosphorylation is blocked by the phosphatase inhibitor cantharidin. The predicted size of the complex suggests that MAPKs and MAPKKs can also interact with additional unknown proteins.

## Materials and methods

### Plant material and growth conditions

Tomato (*Solanum lycopersicum* cv. ‘Rio Grande’), tobacco (*Nicotiana tabacum*), *Nicotiana benthamiana*, and *A. thaliana* plants were grown in AR66L growth chambers (Percival Scientific, Perry, IA, USA) under a 16 h light (110 ± 20 μE m^−2^ s^−1^) and 8 h dark regime. For virus-induced gene silencing (VIGS), plants were grown at 20 °C (light) and 18 °C (dark) for 3 weeks following infiltration, then transferred to 27 °C (light) and 22 °C (dark) for 1 week prior to sampling. Arabidopsis seeds were sterilized briefly in ethanol followed by 5 min in a 20% bleach solution containing 0.5% Tween-20, then washed three times in sterile water. Seeds were vernalized in sterile water for at least 48 h at 4 °C, then germinated on 1⁄2 Murashige and Skoog plates supplemented with 1% (w/v) sucrose for 1 week before being transplanted to soil (MetroMix 360, Sun Gro, Agawam, MA, USA) supplemented with perlite and vermiculite. Arabidopsis plants were grown at 25 °C (light) and 22 °C (dark). The At*MPK3* (At3g45640) T-DNA insertion mutant *mpk3-1* (SALK_151594), the At*MPK6* (At2g43790) T-DNA insertion mutant *mpk6-2* (SALK_073907), and the At*MPK10* (At3g59790) T-DNA insertion mutants *mpk10-1* (SALK_039102; Stanko *et al.*, 2014) and *mpk10-2* (SALK_136149) were obtained from the ABRC (Alonso *et al.*, 2003). *Solanum peruvianum* suspension-cultured cells (Felix and Boller, 1995; Yalamanchili and Stratmann, 2002) were sterilely cultivated in 125 ml Erlenmeyer flasks on an orbital shaker (200 rpm) at room temperature.

### Protein extraction

Plant tissue was flash-frozen in liquid nitrogen and subsequently ground in a chilled mortar. Ground tissue (generally derived from at least two plants per sample) was homogenized with extraction buffer [50 mM HEPES KOH pH 7.6, 1 mM EDTA, 1 mM EGTA, 20 mM β-glycerophosphate, 1 mM Na_3_VO_4_, 1 mM NaF, 20% (v/v) glycerol, 0.5% (w/v) polyvinylpyrrolidone, 2 mM DTT, 1 mM phenylmethylsulfonyl fluoride (PMSF), and 10 μM leupeptin]. The protein concentration was determined using Protein Assay solution (Biorad), and purified BSA (Thermo Scientific, Rockford, IL, USA) was used to generate a standard curve. A 50 μg aliquot of protein for each sample was mixed with 3× SDS gel loading buffer. For gel filtration, tissue was homogenized with a different extraction buffer [50 mM Tris–HCl pH 7.5, 150 mM NaCl, 10 mM MgCl_2_, 2.5 mM EDTA pH 8.0, 25 mM β-glycerophosphate, 10 mM Na_3_VO_4_, 5 mM NaF, 10 μM leupeptin, 1 mM PMSF, 1 mM DTT, 0.1% (v/v) Igepal CA-630 (Sigma-Aldrich, St. Louis, MO, USA), 10% (v/v) glycerol], then centrifuged twice at 14 000 rpm for 10 min at 4 °C, and passed through 0.2 μm filters (Gusmaroli *et al.*, 2004).

### Gel filtration (size-exclusion chromatography)

A 500–1500 μg aliquot of cleared protein extracts was injected onto a Superose 6 10/300 GL column (GE Healthcare, Piscataway, NJ, USA) equilibrated with equilibration buffer (50 mM Tris pH 7.5, 150 mM NaCl, 10 mM MgCl_2_, 2.5 mM EDTA, 25 mM β-glycerophosphate, 10 mM Na_3_VO_4,_ 5 mM NaF). Samples were pumped through the column at a flow rate of 0.5 ml min^–1^ using an ÄKTA Explorer 100 (GE Healthcare). Fractions were collected in 0.5 ml increments for a total volume of 30 ml. Fractions including the HMW complexes (eluting at ~12 ml) and the fractions containing the monomeric proteins (eluting at ~19 ml) were concentrated using StrataClean Resin (Aligent Technologies, La Jolla, CA, USA), then washed briefly with sterile water and eluted from the resin using 10 µl of 3× SDS gel loading buffer. To calibrate the column, a series of marker proteins (Sigma-Aldrich) including albumin (66 kDa, 1.25 mg), β-amylase (200 kDa, 0.5 mg), alcohol dehydrogenase (150 kDa, 0.625 mg), apoferritin (443 kDa, 1.25 mg), and thyroglobin (669 kDa, 1.25 mg) were injected onto the Superose column and the elution profile determined by measuring the UV absorbance at 280, 260, and 220 nm.

### Immunoblot analysis

Proteins were boiled for 5 min before being separated via electrophoresis on a 10% polyacrylamide gel. Separated proteins were transferred to Immobilon-P PVDF membranes (Millipore, Billerica, MA, USA) using a mini transblot electrophoretic transfer cell (Biorad) for 1 h at 100 V in cold transfer buffer (48 mM Tris, 38 mM glycine). The membranes were blocked for 1 h in TBS–Tween-20 [10 mM Tris–HCl pH 7.5, 150 mM NaCl, 0.1% (v/v) Tween-20] containing 5% BSA (Fraction V, Fisher, Waltham, MA, USA). Membranes were washed twice with TBS–Tween-20 and then incubated with the primary antibody overnight at 4 °C with gentle rocking. Blots were washed five times in TBS–Tween-20 and then incubated with an alkaline phosphatase-conjugated secondary antibody (Sigma-Aldrich). After five washes in TBS–Tween-20, blots were analyzed by a chemiluminescence assay by incubating the blots for 5 min in LumiPhos (Pierce, Rockford, IL, USA) or Immuno-Star AP (Biorad) and visualized with HyBlot CL autoradiography film (Denville Scientific Inc., Metuchen, NJ, USA).

Primary antibodies used for immunoblotting were anti-pERK MAPK (Phospho-p44/p42 MAPK, ERK1/2, Thr202/Tyr204, D13.14.4E; Cell Signaling Technology, Danvers, MA, USA) at 1:2500 in TBS–Tween-20 with 5% (w/v) BSA. Anti-pERK specifically recognizes active MAPKs phosphorylated on the threonine (T) and tyrosine (Y) residues within the MAPK-specific TEY phosphorylation motif. Anti-AtMPK6 (Sigma-Aldrich) targets a C-terminal 12 amino acid sequence from Arabidopsis MPK6 (Sigma Product Information) and also recognizes orthologous MAPKs from tomato (MPK1/2) and tobacco (SIPK/Ntf4) (our results). It was used at 1:5000 in TBS–Tween-20. Anti-SlMPK2 antibody was generated against a C-terminal peptide of SlMPK2. The antibody is specific for SlMPK2 and does not cross-react with SlMPK1 (Holley *et al.*, 2003). The anti-rabbit alkaline phosphatase-conjugated secondary antibody (Sigma-Aldrich) was used at 1:20 000 in TBS–Tween-20. To demonstrate equal protein loading and transfer after immunoblotting, proteins on the PVDF membranes were stained using MemCode Reversible Protein Stain Kit for PVDF Membranes (Pierce).

To generate SlMKK4-specific antibodies, the tomato (*S. lycopersicum*) MKK4 cDNA (GenBank accession no. AY691333) was expressed in *Escherichia coli* as an N-terminally His-tagged recombinant protein (Novagen pET28 system) and purified on a nickel column according to standard procedures (Novagen-EMD Millipore). Purified Coomassie G250-stained SlMKK4 protein in polyacrylamide gel was used to raise antibodies in chicken eggs (Cocalico Biologicals, Inc., Reamstown, PA, USA). Pre-bleeds did not contain cross-reacting bands at the predicted molecular weight of SlMKK4 (~37.5 kDa). For immunoblotting of plant leaf extracts, anti-SlMKK4 antibodies (IgY) were detected by incubation with anti-IgY secondary antibodies conjugated to alkaline phosphatase (Sigma-Aldrich) and a subsequent chemiluminescent assay as described above. To demonstrate antibody specificity, anti-SlMKK4 antibodies were pre-incubated with the recombinant MKK4 protein for 45 min prior to being used for immunoblotting.

### Generation of vectors for virus-induced gene silencing

The modified *Tobacco rattle virus* (TRV) vectors, pTRV1 and pTRV2 (Liu *et al.*, 2002), used for virus-induced gene silencing (VIGS) were obtained from S.P. Dinesh-Kumar (UC Davis, CA, USA). A portion of each target gene was cloned into the multiple cloning site (MCS) of the pTRV2 vector. A fragment for each target gene, which was predicted *in silico* to have minimal off-target gene silencing, was amplified from a cDNA library generated from *S. lycopersicum* cv. Rio Grande. For the *VIGS-mpk1/2/3* construct, Sl*MPK1* and Sl*MPK2* (accessions Solyc12g019460 and Solyc08g014420; GenBank accessions nos AY261512 and AY261513) were co-silenced using a 577 bp fragment that was PCR amplified from the MPK1 ORF (primers: MPK1/2F 5'-GCGCGAGCTCCATGGTGGCAGGTTCATTC-3' and MPK1/2R 5'-CGGCGCTCGAGGCTCAGGTCCACGATA CCAT-3'). Tomato *MPK1* and *MPK2* are 89% identical at the nucleotide level. *MPK3* (accession Solyc06g005170; GenBank accession no. AY261514) was specifically silenced using a 406 bp fragment amplified from the 3'-untranslated region (Primers: MPK3F 5'-GGCCGTCTAGAGCATAA GAGAAATCAGTTCTTC-3' and MPK3R 5'-CGCGCGGATCCACACCC AAAACTTCAAAAT GAC-3'). Forward and reverse primers were designed with the flanking restriction site pairs *Sac*I/*Xho*I and *Xba*I/*Bam*HI for ligation into the MCS of pTRV2 for MPK1/2 and MPK3, respectively. The resulting pTRV contains the 577 bp target sequence for MPK1/2 fused to the 406 bp sequence of MPK3 with the restriction site *Kpn*I between the fragments. For the control vector, a 350 bp fragment of green fluorescent protein (*GFP*) was ligated into pTRV2 as previously described (Kandoth *et al.*, 2007). Ligation products were expressed in One Shot DH5α competent cells (Invitrogen/Life Technologies, Grand Island, NY, USA). The sequence of the pTRV vectors was confirmed by DNA sequencing (Eton Biosciences Inc., Durham, NC, USA). The pTRV1 and modified pTRV2 vectors were transfected into *Agrobacterium tumefaciens* strain GV3101. A single colony was selected and inoculated into a liquid culture supplemented with kanamycin (50 μg ml^–1^), rifampicin (50 μg ml^–1^), and gentamycin (30 μg ml^–1^). The culture was incubated overnight at 30 ^o^C with shaking at 250 rpm. To prepare the infiltration media, the overnight culture was centrifuged at 5000 *g* for 10 min at 4 ^o^C. The pellet was washed twice with 10 mM MgCl_2_ and resuspended in infiltration medium [10 mM MgCl_2_, 10 mM MES, and 150 μM acetosyringone (3', 5'-dimethoxy-4'-hydroxyacetophenone, Acros Organics, New Jersey, USA)] to a total OD_600_ of 0.2 and 0.4 for pTRV2 and pTRV1, respectively. Infiltration medium was introduced into the apoplastic space in the cotyledon leaves of 10- to 14-day-old seedlings using a needleless syringe. Plants were sampled 4 weeks after infiltration.

### DNA extraction and Arabidopsis mutant genotyping

Arabidopsis *mpk10* T-DNA insertion lines were tested for mutant allele homozygosity via DNA extraction and subsequent amplification by PCR. DNA was extracted from one rosette leaf of 10- to 14-day-old seedlings by grinding in a 1.5 ml microcentrifuge tube with 0.3 ml of extraction buffer [200 mM Tris–HCl pH 7.5, 250 mM NaCl, 25 mM EDTA pH 8.0, and 0.5% (w/v) SDS]. DNA was precipitated with isopropanol, washed with 70% ethanol, air-dried, and resuspended in sterile water. Extracted DNA was used for PCR analysis using primers specific for areas both upstream and downstream of the predicted T-DNA insertion as well as a primer for the left border of the T-DNA, LBb1.3 (LBb1.3 primer 5'-ATTTTGCCGATTTCGGAAC-3', *mpk10-1* LP 5'-CTAAACACACACCATGCCATG-3', *mpk10-1* RP 5'-GGAAAAGGAAATTCACAGCAG-3', *mpk10-2* LP 5'-TGCAAGTTGTGAATTTGCAAG-3', and *mpk10-2* RP 5'-TTGCTTTGGTTGGGTTTAGTG-3'). Primer sequences were obtained from the Salk Institute Genomic Analysis Laboratory (http://signal.salk.edu/tdnaprimers.2.html).

### Vector construction and transient transformation of *N. benthamiana*

Full-length tomato *MPK1* and *MPK2* (MPK1F 5'-ATGGATGGTTCCGTTCCGC-3', MPK1R 5'-TCACATGCG CTGGTATTCAGGAT-3', MPK2F 5'-ATGATGGTTCAGCTCCG CA-3', and MPK2R 5'-TCACATGTGCTGGTATTCGGGAT-3') were amplified from a Rio Grande cDNA library and cloned into the pCR™/GW/TOPO^®^ vector (Life Technologies). TOPO vector was transformed into competent DH5α *E. coli* cells, and plasmid DNA was isolated from positive transformants. Isolated plasmid was recombined into the Gateway^®^ vectors pEarleyGate 202 (N-terminal FLAG tag) or pEarleyGate 203 (N-terminal c-Myc tag) (Earley *et al.*, 2006) via an LR recombination reaction using LR Clonase^®^ II enzyme mix and transformed by electroporation into One Shot^®^ Mach1™-T1R chemically competent *E. coli* (Life Technologies). Positive transformants were determined by restriction digest and confirmed by sequencing. Confirmed plasmids were transformed into *A. tumefaciens* strain GV3101. One colony was selected and used to start an overnight culture in 3 ml of LB medium. Cells were harvested by centrifugation, washed twice with 10 mM MgCl_2_, and resuspended in infiltration medium (10 mM MgCl_2_, 10 mM MES, and 150 μM acetosyringone) to a final OD_600_ of 0.2–0.4. Using a needleless syringe, infiltration medium was forced into the abaxial side of *N. benthamiana* leaves, which were sampled 3–5 d later.

### FLAG immunoprecipitation

For anti-FLAG immunoprecipitation (IP), GF fractions were collected and samples were pooled from fractions corresponding to HMW or low molecular weight (LMW) fractions (four fractions equaling 2 ml total for each). The IP was performed with an anti-FLAG M2 affinity gel using conditions recommended by the manufacturer (Sigma-Aldrich) with minor variations. Briefly, 1% (v/v) Triton X-100 (Sigma-Aldrich) and 0.5% (v/v) Igepal CA-630 were added to each pooled sample following GF. The affinity gel was prepared as described by the manufacturer using at least 40 μl of resin for each sample. Pooled fraction samples were added to the prepared resin and incubated with tumbling for 2 h at 4 ^o^C. Samples were centrifuged and supernatant removed, then washed three times with TBS buffer (50 mM Tris–HCl,150 mM NaCl, pH 7.4). Samples were eluted with 3× SDS gel loading buffer. Samples were boiled for 5 min then subjected to SDS–PAGE and immunoblot analysis as described above or to Coomassie G250 or R250 staining.

### Tomato suspension culture treatments

For treatments of samples used for immunoblotting (IB), 1.5 ml of *S. peruvianum* suspension-cultured cells were added to each well of 6- or 12-well tissue culture plates (BD Biosciences, San Jose, CA, USA) and shaken on an orbital shaker at 150 rpm under ambient room light and temperature. Cell suspensions were equilibrated for 1 h prior to treatment. For GF experiments, cells (~45 ml) in 125 ml Erlenmeyer flasks were directly treated and placed on an orbital shaker at 200 rpm in ambient room temperature and light. Then 5 ml of cells were collected at each time point for further analysis. Cantharidin (Enzo Life Sciences, Inc., Farmingdale, NY, USA) was dissolved in DMSO and added to the cells for a final concentration of 500 μM. Systemin and flg22 (GenScript, Piscataway, NJ, USA) were dissolved in water and added to the cells at a final concentration of 10 nM. Cells in multiwell plates were irradiated with UV-C (two 15 W UV-C lamps; GE Germicidal Lamp G15T8) at a distance of 10 cm for 5 min while shaking. Cells were removed from the suspension medium using a Büchner filter funnel lined with miracloth, and the cells were immediately flash-frozen in liquid nitrogen.

### In-gel kinase assay

Systemin-treated cell suspension extracts were analyzed by in-gel kinases assays (IGKAs) using myelin basic protein (MBP) as an artificial MAPK substrate as previously described (Stratmann and Ryan, 1997).

## Results

### MAPKs are present in high molecular weight multiprotein complexes

Association of plant MAPK modules with other proteins such as scaffold proteins should lead to the formation of large multiprotein complexes. The molecular weight of the complex consisting of the human KSR scaffold (~100 kDa), the MAPKKK Raf-1 (~73 kDa), the MAPKK MEK1/2 (~43 kDa), and the MAPK ERK1/2 (~43 kDa) is ~260 kDa. The size further increases with additional proteins that interact with the core components. To determine whether plant MAPKs can also be found in HMW multiprotein complexes, we separated plant protein complexes and monomeric proteins by GF. The column used here (Superose 6 10/300 GL) is designed to separate HMW molecules and complexes according to size, but has a poor resolution for LMW proteins, which tend to elute over a range of fractions. To better compare GF elution profiles in the figures shown, the elution volumes in increments of 0.5 ml fractions eluting from the GF column are indicated below the profiles, and the peak elution of molecular weight marker proteins, which provide a size estimate of the eluted protein complexes, are shown above the profiles.

**Fig. 1.**
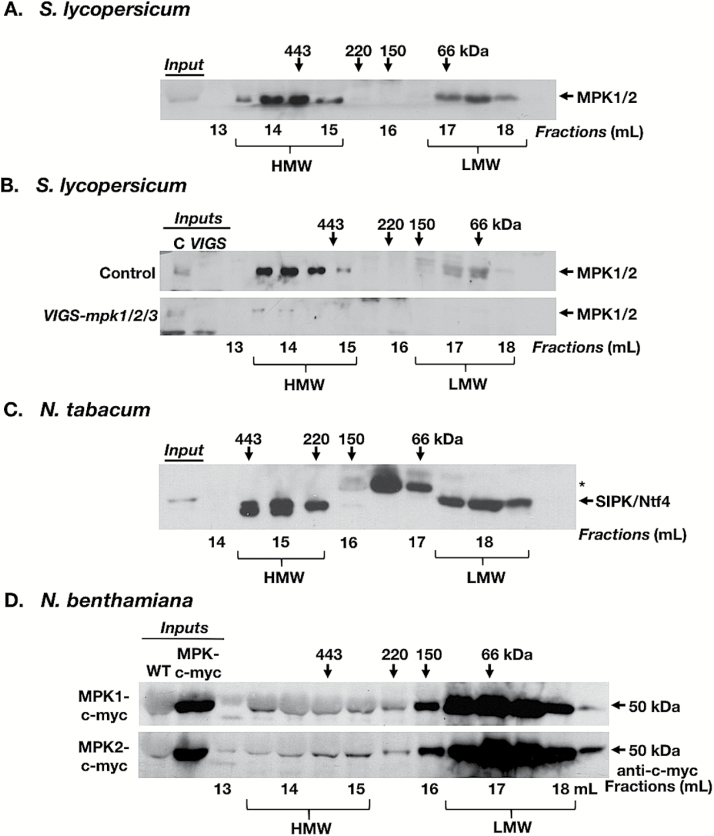
Identification of a MAP kinase-containing multiprotein complex in tomato and tobacco. Total protein was extracted from leaf tissue and separated by GF. Eluate was collected in 0.5 ml fractions, concentrated, and analyzed by immunoblotting (IB). Only fractions representing 13–18.5 ml are shown (numbers underneath the immunoblots). The numbers above the panels indicate the peak elution of molecular weight standards in kDa. Input samples represent 30 μg of total protein extracted from leaf tissue and represent the same protein extracts that were used for GF. (A) GF/IB analysis of leaf extracts (1.5 mg of total protein) from tomato plants (*S. lycopersicum*). SlMPK1/2 were detected using anti-AtMPK6 antibody. (B) GF/IB analysis of leaf extracts (1.5 mg of total protein) from tomato plants in which *MPK1*, *MPK2*, and *MPK3* were co-silenced via VIGS (*VIGS-mpk1/2/3*) and from control plants. SlMPK1/2 were detected using anti-AtMPK6 antibody. Inputs represent 30 µg of total protein from control plants (C) or *VIGS-mpk1/2/3* plants (VIGS). (C) GF/IB analysis of leaf extracts (1.2 mg of total protein) from tobacco plants (*N. tabacum*). SIPK and Ntf4 were detected using anti-AtMPK6 antibody. (D) GF/IB analysis of leaf extracts (1 mg of total protein) from *N. benthamiana* plants transiently overexpressing *35S:SlMPK1-c-myc* or *35S:SlMPK2-c-myc*, probed with anti-c-myc antibody. Inputs represent 30 μg of total protein from non-infiltrated *N. benthamiana* leaves (WT) or from leaves overexpressing fusion proteins (MPK-c-myc). The shift towards higher molecular weight (up to 150 kDa) in the LMW fractions is probably an artifact caused by the high amounts of tagged MPK1/2 protein due to overexpression. HMW, MAPK-containing high molecular weight fractions; LMW, MAPK-containing low molecular weight fractions. (A) is representative of seven, (B) of three, (C) of four, and (D) of one independent experiment, with each sample containing tissue from at least two different plants. *Unrelated protein that cross-reacts with anti-AtMPK6 antibodies.

We probed concentrated proteins from GF fractions by IB with an anti-AtMPK6 antibody, which was generated against *A. thaliana* MPK6 (AtMPK6). Using VIGS, we showed that this antibody also recognizes orthologs in tomato (*S. lycopersicum*) (SlMPK2 and/or SlMPK1) (Fig. 1B). Based on our IB data (Figs 1C, 5) and the phylogenetic relationship among AtMPK6 orthologs (see Supplementary Fig. S1 at *JXB* online), this antibody probably recognizes AtMPK6 orthologs in the wild tomato species *S. peruvianum* (SpMPK1 and/or SpMPK2), and in tobacco (SIPK and/or NTF4). These MAPKs all have an apparent molecular weight of ~48 kDa. No bands corresponding to the lower apparent molecular weight of AtMPK3 and AtMPK4 orthologs were detected in these species, indicating that the anti-AtMPK6 antibody specifically recognizes MPK6 orthologs.

The GF elution profile from *S. lycopersicum* leaf extracts is biphasic. An ~48 kDa band was prominent in <100 kDa GF fractions (fractions ≥17 ml) and represents monomeric MPK1/2 (Fig. 1A). Based on the resolution of the Superose column, it cannot be excluded that these fractions contain MPK6 associated with another relatively small protein, but, as a working hypothesis, we will refer to the MPKs in LMW fractions (<100 kDa) as monomeric MPKs. In addition, we detected an ~48 kDa band in HMW GF fractions (Fig. 1A, fractions 13.5–15), corresponding to protein complex sizes between 300 kDa and 550 kDa.

In tomato plants, we used VIGS to reduce the expression of three highly homologous MAPK genes, Sl*MPK1*, Sl*MPK2*, and Sl*MPK3* (Holley *et al.*, 2003; Kandoth *et al.*, 2007). Sequences corresponding to the MAPKs were inserted into the genome of the TRV encoded on a binary vector, followed by transformation of the manipulated TRV vector into *A. tumefaciens* and delivery into plants by cotyledon infiltration. Four weeks later, plants were analyzed for silencing of MAPK genes. In leaf extracts of negative control plants that were infiltrated with TRV containing a fragment of *GFP*, SlMPK1/2 was detected by the anti-AtMPK6 antibody in HMW and LMW fractions (Fig. 1B, upper panel), similarly to uninfected plants (Fig. 1A). In contrast, in *VIGS-mpk1/2/3* plants, only low amounts of SlMPK1/2 protein were detected (Fig. 1B, lower panel). Additionally, an antibody against SlMPK2 (Holley *et al.*, 2003) also recognized a band in HMW and LMW GF fractions from control plants, and this band was reduced in extracts from *VIGS-mpk1/2/3* plants (see Supplementary Fig. S2). An anti-SlMPK3 antibody suitable for IB was not available, and a frequently used anti-AtMPK3 antibody (Sigma-Aldrich) does not recognize the tomato ortholog SlMPK3.

GF/IB analysis with anti-AtMPK6 antibody also detected ~48 kDa proteins in HMW GF fractions of leaf extracts from tobacco (*N. tabacum*) (~200–450 kDa; Fig. 1C) and *N. benthamiana* (Supplementary Fig. S3A; 300–500 kDa), as well as in extracts from *S. peruvianum* suspension cell cultures (~300–450 kDa) (Fig. 4). The double band in *N. tabacum* HMW fractions (Fig. 1C) may represent SIPK and NTF4, which are ordinarily not separated by SDS–PAGE (Ren *et al.*, 2006). Figure 4 shows that the two bands do not represent the phosphorylated and unphosphorylated forms of a SIPK/NTF4; however, they may have additional post-translational modifications that change their electrophoretic mobility. This could also be the case for the double band (MPK1/2) in LMW fractions of *S. lycopersicum* (Fig. 1B), although this pattern was not reproducible.

To demonstrate further the presence of MPKs in HMW GF fractions, *N. benthamiana* plants were transiently transformed with *A. tumefaciens* containing a c-myc-tagged *SlMPK1* or *SlMPK2.* Both c-myc-tagged SlMPK1 and SlMPK2 proteins were incorporated into a HMW protein complex (Fig. 1D). The GF elution profiles for both the c-myc-tagged and the FLAG-tagged MAPKs was somewhat different as compared with GF profiles of untagged MAPKs, perhaps as an artifact of overexpression. Tagged MAPKs were also present in the fractions between the LMW and HMW fractions (100–300 kDa). In addition, transient expression of a FLAG-tagged *SlMPK1* in *N. benthamiana* leaves and subsequent GF/IB of leaf extracts with an antibody against the FLAG tag resulted in detection of an ~48 kDa band in HMW and LMW fractions. However, there was also a cross-reacting band of a similar molecular weight present in untransformed control plants (Supplementary Fig. S3B), although the signal was much weaker than in extracts from leaves expressing SlMPK1-FLAG. In addition, FLAG-tagged proteins in HMW and LMW fractions from leaf extracts were first immunoprecipitated with an anti-FLAG antibody to enrich the proteins, then analyzed by IB with the same anti-FLAG antibody. Transformed plants contained an ~48 kDa FLAG-tagged protein, which was more abundant in LMW GF fractions, but also clearly present in HMW GF fractions (Supplementary Fig. S3C).

**Fig. 2.**
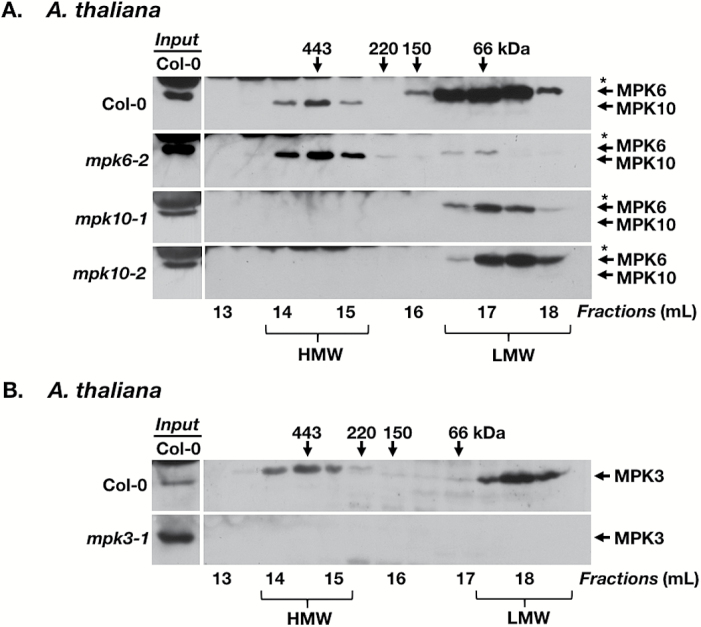
Identification of a MAP kinase-containing multiprotein complex in *Arabidopsis thaliana*. (A) GF/IB analysis of leaf extracts from *A. thaliana* ecotype Columbia-0 (Col-0, 1.5 mg of total protein), and the null mutants *mpk6-2* (1.5 mg), *mpk10-1* (0.5 mg), and *mpk10-2* (0.5 mg). AtMPK6 and AtMPK10 were detected using anti-AtMPK6 antibody. (B) GF/IB analysis of leaf extracts from *A. thaliana* ecotype Col-0 (1.5 mg of total protein) and the *mpk3-1* null mutant (0.75 mg). AtMPK3 was detected using anti-AtMPK3 antibody. Note: the Col-0 column on the left shows extracts (30 µg of total protein) from Col-0 as a reference for MPK6 (A) or MPK3 (B). These samples were analyzed on the same blot as the corresponding GF fractions, but additional bands were removed to adjust the elution profiles among panels. This is indicated by the white line that separates reference samples and GF fractions. For the two *mpk-10* rows, shorter exposures for the Col-0 reference lane are shown as compared with the GF fractions. Numbers above and below the immunoblots are as described in Fig. 1. Immunoblots for Col-0 and *mpk6-2* represent four independent experiments; and those for the two independent *mpk10* mutant lines were performed once for each line.

In *A. thaliana*, many MAPK mutant lines are available, which allowed us to ascertain specifically which MAPK components were present in the HMW versus the LMW fractions. In leaves of Arabidopsis (Fig. 2A) a band with a size range between 300 kDa and 450 kDa was detected in HMW GF fractions after probing immunoblots of GF fractions with the anti-AtMPK6 antibody. However, the apparent molecular weight of the protein in HMW fractions is lower (indicating a size of ~45 kDa) than the molecular weight of the proteins in LMW fractions (~48 kDa). Importantly, this band was still present in the *mpk6-2* null mutant, whereas the 48 kDa band in the LMW GF fractions was absent. A very weak band at ~45 kDa persisted in LMW fractions 16.5 and 17 (Fig. 2A), indicating that the anti-AtMPK6 antibody recognizes an additional protein, which is present as a monomer but more abundant in a HMW protein complex. The C-terminal 12 amino acid peptide sequence of AtMPK6 against which the anti-AtMPK6 antibody was raised (REALAFNPEYQQ) shares eight amino acids with a sequence close to the C-terminus of the highly homologous AtMPK10 (EALAFNPE) (Supplementary Fig. S1). In two different *mpk10* T-DNA insertional mutants (for characterization, see Supplementary Fig. S4), the 45 kDa bands detected by the anti-AtMPK6 antibody in the HMW GF fractions were absent, while the 48 kDa band (MPK6) in the LMW fractions persisted (Fig. 2A). Together, this indicates that in Arabidopsis, MPK10 rather than MPK6 associates with a HMW protein complex and that the majority of the monomeric MPK protein in the LMW fractions is MPK6 protein. MPK10 is a closely related paralog of MPK6 (Supplementary Fig. S1) and plays a role in polar auxin transport (Stanko *et al.*, 2014). A role in stress responses has not been investigated. In Col-0, the elution profile of MPK6 in LMW fractions ranges from fraction 16 (150 kDa) to fraction 18 (<66 kDa). In the *mpk10* mutants, a small fraction of MPK6 protein elutes in fraction 16.5 and the majority in fractions representing molecular weights of <66 kDa, which is more in line with elution profiles of extracts from solanaceous plants shown in Fig. 1. The differences in the elution profiles of Col-0 and *mpk10* mutants are most probably due to different amounts of total protein analyzed confounded by the low resolution of the column for LMW proteins, and do not necessarily indicate an association of Arabidopsis MPK6 with other proteins.

Besides MPK10, MPK3 also associated with a HMW protein complex in Arabidopsis leaves (Fig. 2B), as shown by GF followed by IB with an anti-AtMPK3 antibody. Like MPK10, MPK3 protein was detected in HMW GF fractions ranging from 300 kDa to 450 kDa. All MPK3 bands were absent in the *mpk3-1* null mutant.

### MKK4 is part of a HMW multiprotein complex

**Fig. 3.**
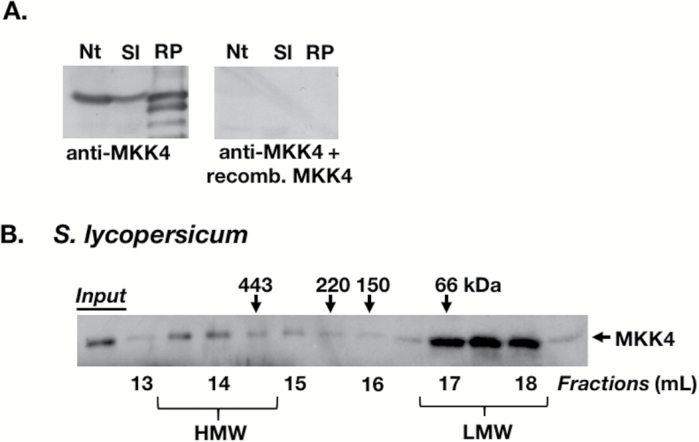
*Solanum lycopersicum* MKK4 associates with a multiprotein complex. (A) Extracts from *N. tabacum* (Nt) and *S. lycopersicum* (Sl) leaves as well as affinity-purified recombinant SlMKK4 protein (RP) were analyzed by IB using either anti-SlMKK4 antiserum (left panel) or anti-SlMKK4 antiserum pre-incubated with recombinant MKK4 protein (right panel). Note that the lane showing purified MKK4 protein contains MKK4 degradation products. The binding of free MKK4 by the antibody prevented it from recognizing MKK4 on the immunoblot membrane. The MKK4 ortholog from *N. tabacum* (MKK9; accession no. NP_001311802) shares 88% identity with SlMKK4 at the amino acid level. Without a stretch of 16 amino acids that is absent in NtMKK9, the identity is 93%. This explains why the anti-SlMKK4 antibody recognizes a protein in *N. tabacum*, presumably NtMKK9. (B) GF/IB analysis of leaf extracts (1.5 mg) from *S. lycopersicum* plants. SlMKK4 was detected using anti-SlMKK4 antibody. Numbers above and below the immunoblots are as described in Fig. 1. All immunoblots represent a minimum of two independent experiments.

If MAPKs associate with a protein complex to facilitate interactions with specific activating proteins such as MAPKKs, then these kinases may also be present in HMW protein complexes. We generated a specific antibody against tomato MKK4 (D-group of MAPKKs) (Fig. 3A). In tomato, MKK4 and MKK2 are the upstream MAPKKs that activate the MAPKs MPK1 and MPK2 (Pedley and Martin, 2004). We probed GF fractions of tomato leaf tissue with this antibody. MKK4 showed a biphasic elution pattern from the GF column with a major peak in the LMW fractions at <100 kDa and a minor peak in HMW fractions at >440 kDa (Fig. 3B). The MKK4 band was also detectable in fractions between the two peaks, unlike the MAPK bands. This demonstrates that MKK4 is also associated with HMW multiprotein complexes.

### Phosphorylated MAPKs do not normally associate with a HMW multiprotein complex

The antibodies described so far do not distinguish between active and inactive MAPKs that are or are not phosphorylated on the threonine and tyrosine of the TEY MAPK phosphorylation motif. To test whether TEY-phosphorylated MAPKs associate with a HMW multiprotein complex, we probed GF fractions with anti-pERK antibody. This antibody was raised against the dually phosphorylated TEY motif of human p44 MAPK. Anti-pERK is widely used to demonstrate the presence of phosphorylated MAPKs across kingdoms, including plants (Hann *et al.*, 2014; Buscà *et al.*, 2015).

**Fig. 4.**
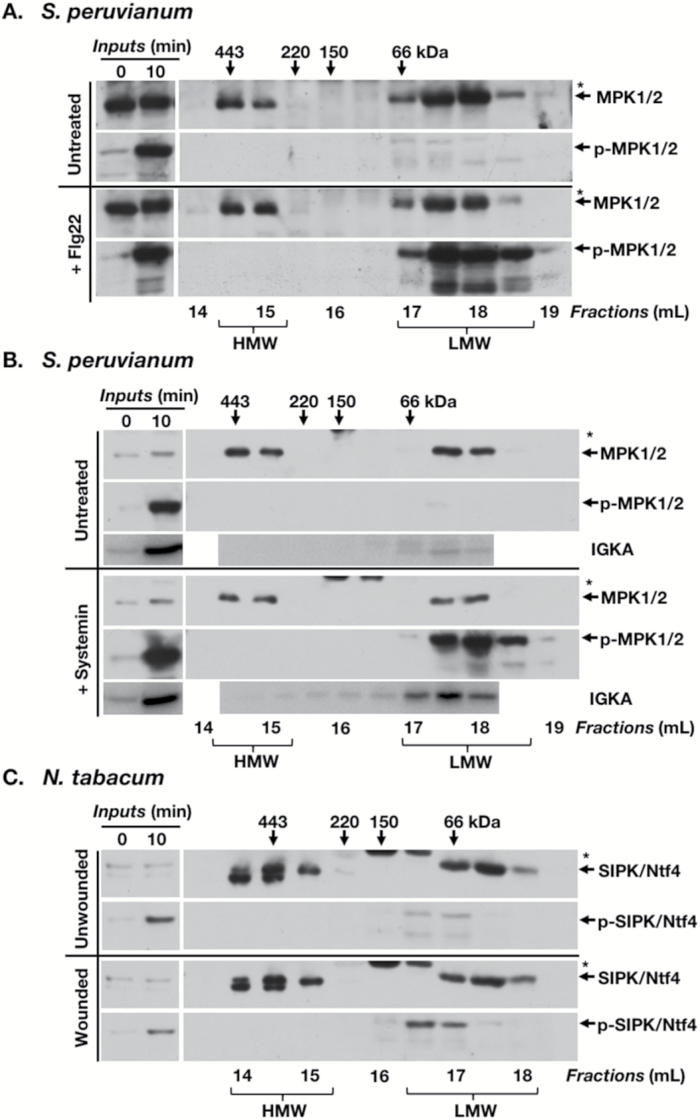
Active phosphorylated MPK1/SIPK and MPK2/Ntf4 do not normally associate with a multiprotein complex in tomato and tobacco. *Solanum peruvianum* suspension-cultured cells were treated with 10 nM flg22 or 10 nM systemin. Total protein was extracted from suspension cells 0 and 10 min after elicitor treatment. Inputs represent 30 μg of total protein extracted from cells collected at the times indicated after treatment. Numbers above and below immunoblots are as described in Fig. 1. (A) GF/IB analysis of extracts (0.9 mg of total protein) from untreated suspension cells or cells treated with flg22, probed with anti-AtMPK6 antibody for the detection of total MPK1/2 and anti-pERK antibody for the detection of phosphorylated MPK1/2 (p-MPK1/2). (B) The same as (A) but after treatment with systemin (0.75 mg of total protein). The additional panel shows enzymatic activity of MPK1/2 as determined by in-gel kinase assays (IGKAs). Total protein (1.5 mg) was separated by GF and each fraction was analyzed by IGKA. Signals represent ^32^P-phosphorylated myelin basic protein. (C) GF/IB analysis of extracts (1.2 mg of total protein) from tobacco leaves left untreated or wounded and sampled 10 min later, probed with anti-AtMPK6 antibody for the detection of SIPK/Ntf4 and anti-pERK antibody for the detection of phosphorylated SIPK/Ntf4 (p-SIPK/Ntf4). (A) and immunoblots in (B) are representative of four independent experiments; IGKAs in (B) and (C) of two independent experiments. For tobacco, similar results were seen for 180 min after wounding (Supplementary Fig. S5B). *Unrelated protein that cross-reacts with anti-AtMPK6 antibodies.

When *S. peruvianum* suspension-cultured cells were treated for 10 min with the bacterial microbe-associated molecular pattern (MAMP) flg22, the damage-associated molecular pattern (DAMP) systemin (Fig. 4A, B), or irradiated with UV-C radiation (Supplementary Fig. S5A), the anti-pERK antibody detected phosphorylated MAPKs (SpMPK1/2 and SpMPK3) only in the LMW fractions after treatment. Probing with anti-AtMPK6 shows that SpMPK1/2 is present in both LMW and HMW fractions from extracts of both treated and untreated suspension-cultured cells (Fig. 4A, B). This indicates that only unphosphorylated (i.e. inactive) SpMPK1/2 is present in the HMW multiprotein complex. The situation was similar in leaf tissue of tobacco, where wound-induced phosphorylation of SIPK was only detected in LMW GF fractions (Fig. 4C).

According to the manufacturer, anti-pERK may also recognize MAPKs singly phosphorylated at the T of the TEY motif. Singly phosphorylated MAPKs are not enzymatically active (Robbins *et al.*, 1993). In order to test whether enzymatically active MAPKs are also confined to LMW GF fractions, IGKAs were performed on extracts from *S. peruvianum* suspension cells treated with systemin (Fig. 4B) and from tomato plants 10 min after wounding (Supplementary Fig. S5C). These results confirm the IB phosphorylation assays and together demonstrate that phosphorylated and enzymatically active MAPKs are only present in LMW GF fractions.

### In the presence of a phosphatase inhibitor, phosphorylated MAPKs do associate with a HMW multiprotein complex

Using IGKAs and IB with anti-pERK antibodies, we were unable to detect phosphorylated MAPKs in HMW GF fractions in untreated and in stimulated *S. peruvianum* cells (Fig. 4). Notably, the ratio of complex-associated MAPKs to monomeric MAPKs remained unchanged up to 180 min after treatment with systemin or flg22 (Fig. 4; Supplementary Fig. S5D). To explore further the phosphorylation state of complex-associated MAPKs, we treated *S. peruvianum* cells with the phosphatase inhibitor cantharidin.

**Fig. 5.**
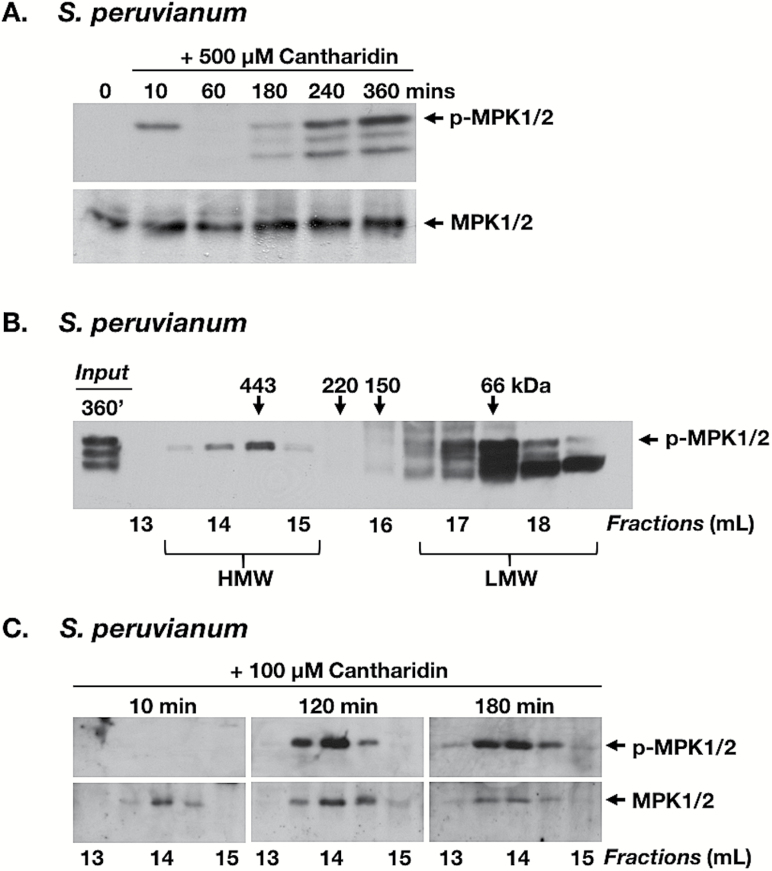
Phosphorylated MPK1 and MPK2 associate with a multiprotein complex after treatment with a phosphatase inhibitor. *Solanum peruvianum* cells were treated with the phosphatase inhibitor cantharidin and sampled at the times indicated. (A) IB analysis of cell extracts (30 µg) from suspension cells sampled at the times indicated after 500 μM cantharidin treatment, probed with anti-pERK antibody (upper panel) and anti-AtMPK6 antibody (lower panel) for the detection of phosphorylated and total MPK1/2, respectively. (B) GF/IB analysis of cell extracts (1.5 mg total protein) from suspension cells sampled at 360 min after 500 μM cantharidin treatment, probed with anti-pERK antibody for the detection of phosphorylated MPK1/2. Input represents 30 μg of total protein. (C) GF/IB analysis of cell extracts (1.5 mg of total protein) from suspension cells sampled at 10, 120, and 180 min after treatment with 100 μM cantharidin, probed with anti-pERK antibody for the detection of phosphorylated MPK1/2 (upper panel). Immunoblots were stripped and reprobed with anti-AtMPK6 antibody for the detection of total MPK1/2 (lower panel). Only HMW fractions were analyzed (13–15 ml). Numbers above and below the immunoblots are as described in Fig. 1. Similar results were obtained in at least four independent experiments.

Cantharidin is a moderate (nM to high µM range) inhibitor of several serine/threonine protein phosphatases, notably PP2A (Pereira *et al.*, 2011). Treatment of suspension-cultured cells with cantharidin induced a biphasic time course of MPK6 activity with a rapid early peak at 10 min and return to background levels, followed by a second slower increase starting 60–120 min after treatment and lasting for at least 6 h. During this second phase, two additional bands (~46 kDa and ~44 kDa) were detected by anti-pERK, most probably representing increased activity of SpMPK3 and the tomato ortholog of AtMPK4 (Fig. 5A). Extracts from cantharidin-treated cells were analyzed by GF/IB with anti-pERK and anti-AtMPK6 antibodies. Anti-pERK detected a phosphorylated 48 kDa MAPK in HMW and LMW fractions at 120–360 min after cantharidin treatment (Fig. 5B, C), but not in untreated cells (Fig. 4) and not during the first phase of cantharidin-induced MAPK activity at 10 min (Fig. 5A, C). Reprobing the immunoblots with anti-AtMPK6 antibody confirmed the presence of SpMPK1/2 in the same fractions that contained the phosphorylated 48 kDa MAPKs. No phosphorylated 46 kDa and 44 kDa MAPKs were detected in HMW fractions (Fig. 5B, C). These results indicate that cantharidin inhibition of MAPK dephosphorylation by phosphatases results in increased SpMPK1/2 activity over time and in association of phosphorylated SpMPK1/2 with a HMW protein complex.

## Discussion

A number of more or less complete MAPK signaling pathways and networks have been described in plants, and the function of specific MAPK pathway components has been revealed. However, it is still poorly understood how MAPK modules are organized in response to an input signal. Several proteins have been identified in plants that interact with components of the MAPK pathways including phosphatases, 14-3-3 proteins, and MAPK substrate proteins (Andreasson and Ellis, 2010; Oh *et al.*, 2010; Rodriguez *et al.*, 2010), but it is largely unknown how, when, and where they interact with a MAPK module. In animal cells, MAPK-containing multiprotein complexes have been well characterized. With the exception of the recently discovered RACK1 complex, no such large complexes have been discovered in plant cells thus far. Examples of additional but smaller plant MAPK complexes include a nuclear complex consisting of MPK4, the MPK4 substrate MKS1, and the transcription factor WRKY33 (Qiu *et al.*, 2008), and a potentially larger complex which contains OMTK1, an alfalfa MAPKKK. OMTK1 interacts directly with the MAPK MMK3, combining in one protein the kinase and the scaffolding function (Nakagami *et al.*, 2004), and potentially giving rise to a larger complex. Here we provide evidence that certain plant MAPKs and MAPKKs associate with stable HMW multiprotein complexes. While the nature of the other constituents of these complexes remains to be determined, it is likely that they include the proteins known to interact with the members of a MAPK module as mentioned above.

The apparent size of the MAPK-containing complexes we detected by GF/IB varies depending on the plant species, the amount of protein loaded onto the GF column, and whether proteins from leaf material or suspension-cultured cells were analyzed. On average, complex-associated MAPKs eluted in fractions corresponding to molecular weights of 300–550 kDa. The peak elution profile for the tomato MAPKK MKK4 was similar to tomato MPK1/2 elution profiles, but additional fractions contained small amounts of MKK4 that did not contain MPK1/2 protein. There is precedent that different components of a multiprotein complex may show slightly different elution profiles (Dohmann *et al.*, 2005; Gusmaroli *et al.*, 2007; Hind *et al.*, 2011). A biphasic elution profile was also obtained for c-myc-tagged MPK1 and MPK2 proteins, but the specific profiles were slightly different for each tagged MAPK and they were detected in all fractions tested. The elution patterns for MPK1-c-myc and MPK2-c-myc are similar, but the highest levels of MPK1-c-myc elute in fraction 13.5 and of MPK2-c-myc in fractions 14.5 and 15. This is probably experimental variation, but it cannot be excluded that both very similar MAPKs associate with different multiprotein complexes. Overexpression resulted in strongly increased amounts of MAPK protein in LMW fractions, but not in HMW fractions, as compared with proteins from wild-type plants and cells. We hypothesize that the amount of MAPKs that associate into a multiprotein complex is limited by other proteins (e.g. scaffolding proteins). Since such proteins are not up-regulated in MPK1/2-overexpressing plants, the amount of MPK1/2 in HMW fractions is not expected to increase, and the majority of the overexpressed proteins elute as monomeric proteins in LMW fractions. Since tagged MPK1/2 are present in HMW fractions, they probably occupy the same binding sites within the multiprotein complex as the native MPK1/2. The shift towards higher molecular weight (up to 150 kDa) in the LMW fractions is likely to be an artifact caused by the high amounts of tagged MPK1/2 protein due to overexpression.

Although the sizes of MAPKKK proteins can be highly variable (Lafleur *et al.*, 2015), a complex consisting of the three components of the MAPK module is predicted to be ~200 kDa on average, indicating that additional proteins may be present in the HMW complexes we detected. The ratio of LMW (monomeric) to HMW MAPKs in GF fractions was somewhat variable between systems, but in general a significant amount of total MAPK protein eluted in the HMW fractions, with the exception of Arabidopsis MPK10. The KSR/ERK/MEK/B-RAF-containing complex from mouse brain tissue showed elution profiles similar to those shown here for plants (Nguyen *et al.*, 2002). ERK was detected in GF fractions corresponding to molecular weights ranging from ~50 kDa to 500 kDa. The highest levels of ERK protein were detected in fractions corresponding to a molecular weight of ~ 200–400 kDa, but, in contrast to the GF profiles in plants, the relative amount of ERK in LMW fractions was lower (Nguyen *et al.*, 2002). In Arabidopsis, we observed that the majority of MPK10 is associated with a HMW complex, similar to ERK in animals.

It was surprising to find that Arabidopsis MPK6 did not associate with a HMW complex, in contrast to its orthologs in tomato and tobacco. Instead, we demonstrated that the closely related Arabidopsis MPK10 associates with a HMW complex. MPK10 is the closest paralog of MPK6 and MPK3 in Arabidopsis (72% and 65% identity at the amino acid level, respectively). Thus it is not as similar to MPK6 as is tomato MPK1 to MPK2 (95.4% identity) or tobacco SIPK to Ntf4 (93.6% identity) (Supplementary Fig. S1) (Holley *et al.*, 2003; Ren *et al.*, 2006; Kong *et al.*, 2012). Therefore, MPK6 and MPK10 may be a product of an earlier gene duplication event. A detailed study demonstrated that MPK10 has a highly localized expression in leaves which supports its function in the development of leaf venation, and a role in polar auxin transport (Stanko *et al.*, 2014). MPK10 was also shown to interact with MKK2 (Stanko *et al.*, 2014) while MPK6 interacts with other MAPKKs. Since MPK6 functions in other developmental processes such as stomatal differentiation (Wang *et al.*, 2007; Lampard *et al.*, 2008) and stress responses, and a function of MPK10 in plant stress responses has not been reported, MPK10 and MPK6 seem to have undergone substantial neofunctionalization although they are close paralogs. However, too little is known about MPK10 to explain why MPK10 but not MPK6 associates with HMW complexes. On the other hand, AtMPK3 was clearly shown to associate with a HMW complex as no MPK3 protein was detectable in the *mpk3* null mutant.

It has been demonstrated previously that tomato MKK4 functions upstream of MPK1/2 (Pedley and Martin, 2004), and we confirmed this activity using a protoplast transient transformation system in which MKK4 phosphorylates MPK1/2, but not MPK3 (data not shown). Our data are consistent with MKK4 and MPK1/2 being present in the same multiprotein complex. However, it cannot be excluded that they associate with separate complexes of a similar molecular weight.

In animal cells, some phosphatases associate with multiprotein complexes organized by scaffolds such as paxillin, KSR, and JIP1 (Willoughby *et al.*, 2003; Deakin and Turner, 2008; Shaw and Filbert, 2009). In plants, different types of phosphatases can dephosphorylate and interact *in vivo* with MPK6, including: the dual-specificity MAPK phosphatases MKP1, MKP2, DsPTP1, PHS1, and IBR5; the PP2C-type Ser/Thr phosphatases AP2C1/2/3/4 and ABI1; and the Tyr-specific phosphatase AtPTP1 (Leung *et al.*, 2006; Schweighofer *et al.*, 2007; Bartels *et al.*, 2009; Lumbreras *et al.*, 2010; Umbrasaite *et al.*, 2010). In the presence of cantharidin, tomato MPK1/2 activity gradually increased after 60 min, presumably due to inhibition of one or more MAPK phosphatases. Cantharidin is a PP2A-type phosphatase inhibitor, and thus far no such phosphatase has been characterized as a MAPK-inactivating phosphatase in plants. Therefore, it cannot be excluded that cantharidin activates MAPKs indirectly via inhibition of unknown phosphatases, which dephosphorylate other components of the MAPK pathways such as MAPKKs or MAPKKKs. In animal cells, PP2A can inhibit the activity of the MAPK ERK without dephosphorylation of the TEY motif (Letourneux *et al.*, 2006). Regardless, cantharidin-activated MPK1/2 does associate with a HMW protein complex, suggesting that phosphatases regulate association of MAPKs with the complex.

The MAPK-containing multiprotein complex in *S. peruvianum* cells may operate in a similar manner to the RACK1 complex in Arabidopsis. Arabidopsis RACK1 binds to the MAPKKK MEKK1, the MAPKKs MKK4 and MKK5, and the two MAPKs MPK6 and MPK3. This complex is fully assembled in the absence of a stimulus, and, in response to treatment of Arabidopsis with a bacterial protease IV elicitor, the complex disassembles, thus releasing the active phosphorylated forms of MKK4/5 and MPK3/6, which can then activate defense gene expression (Cheng *et al.*, 2015; Meng *et al.*, 2015; Su *et al.*, 2015). In addition, RACK1 appears constitutively to interact with the G-protein Gβ. Gβ and RACK1 did not play a role in the activation of the same MAPK module by the bacterial elicitor flg22, thus demonstrating how a scaffold protein confers signaling specificity (Cheng *et al.*, 2015; Su *et al.*, 2015). This result also indicated that only a portion of the MPK3/6 proteins resides in the RACK1 complex, while the remaining proteins are presumably either unbound or associated with other proteins. The latter is also true for the MAPKs we found associated with a HMW multiprotein complex (MPK1/2 and SIPK/NTF4), with the exception of MPK10, which is mainly associated with a HMW complex.

In tomato cells, our results suggest that a phosphatase inactivates MAPKs before or while they associate with the complex. This would result in a pool of MAPKs which can be re-activated by incoming signals. Blocking this phosphatase with a phosphatase inhibitor such as cantharidin results in aberrant association of active MAPKs with the complex. Interaction between inactive MAPKs and other complex-associated proteins may be destabilized upon MAPK phosphorylation by either a complex-associated or an unbound MAPKK. It is unclear if the phosphatase, while controlling the association/dissociation of MAPKs with/from the complex, is a constituent of the complex itself. This working model is speculative but can be tested, pending the identification of the components of the multiprotein complex. Our data suggest that the complex remains relatively stable, which should facilitate the identification of additional components.

## Supplementary data

Supplementary data are available at *JXB* online.

Fig. S1. Phylogenetic analysis of MPK3, 6, 10 and orthologs.

Fig. S2. Reduction of monomeric and complex-associated SlMPK2 in *VIGS-mpk1/2/3* plants.

Fig. S3. MAP kinase-containing multiprotein complexes in *N. benthamiana*.

Fig. S4. Characterization of Arabidopsis T-DNA insertion lines *mpk10-1* and *mpk10-2*.

Fig. S5. GF/IB analyses showing that unphosphorylated MPKs associate with a multiprotein MAPK complex in response to UV-C, wounding, and systemin.

supplementary-figures-S1-S5

## Abbreviations:

GF: gel filtration
HMW: high molecular weight
IB: immunoblotting
IGKA: in-gel kinase assay
LMW: low molecular weight
VIGS: virus-induced gene silencing.

## References

[CIT0001] Alonso JM, Stepanova AN, Leisse TJ, et al 2003 Genome-wide insertional mutagenesis of *Arabidopsis thaliana*. Science 301, 653–657. 12893945 10.1126/science.1086391

[CIT0002] Andreasson E, Ellis B 2010 Convergence and specificity in the Arabidopsis MAPK nexus. Trends in Plant Science 15, 106–113. 20047850 10.1016/j.tplants.2009.12.001

[CIT0003] Bartels S, Anderson JC, González Besteiro MA, Carreri A, Hirt H, Buchala A, Métraux JP, Peck SC, Ulm R 2009 MAP kinase phosphatase1 and protein tyrosine phosphatase1 are repressors of salicylic acid synthesis and SNC1-mediated responses in Arabidopsis. The Plant Cell 21, 2884–2897. 19789277 10.1105/tpc.109.067678 PMC2768924

[CIT0004] Bartels S, González Besteiro MA, Lang D, Ulm R 2010 Emerging functions for plant MAP kinase phosphatases. Trends in Plant Science 15, 322–329. 20452268 10.1016/j.tplants.2010.04.003

[CIT0005] Beck M, Komis G, Müller J, Menzel D, Samaj J 2010 Arabidopsis homologs of nucleus- and phragmoplast-localized kinase 2 and 3 and mitogen-activated protein kinase 4 are essential for microtubule organization. The Plant Cell 22, 755–771. 20215588 10.1105/tpc.109.071746 PMC2861451

[CIT0006] Bigeard J, Colcombet J, Hirt H 2015 Signaling mechanisms in pattern-triggered immunity (PTI). Molecular Plant 8, 521–539. 25744358 10.1016/j.molp.2014.12.022

[CIT0007] Buscà R, Christen R, Lovern M, Clifford AM, Yue JX, Goss GG, Pouysségur J, Lenormand P 2015 ERK1 and ERK2 present functional redundancy in tetrapods despite higher evolution rate of ERK1. BMC Evolutionary Biology 15, 179. 26336084 10.1186/s12862-015-0450-x PMC4559367

[CIT0008] Champion A, Picaud A, Henry Y 2004 Reassessing the MAP3K and MAP4K relationships. Trends in Plant Science 9, 123–129. 15003235 10.1016/j.tplants.2004.01.005

[CIT0009] Cheng Z, Li JF, Niu Y, et al 2015 Pathogen-secreted proteases activate a novel plant immune pathway. Nature 521, 213–216. 25731164 10.1038/nature14243 PMC4433409

[CIT0010] Deakin NO, Turner CE 2008 Paxillin comes of age. Journal of Cell Science 121, 2435–2444. 18650496 10.1242/jcs.018044 PMC2522309

[CIT0011] Dohmann EM, Kuhnle C, Schwechheimer C 2005 Loss of the CONSTITUTIVE PHOTOMORPHOGENIC9 signalosome subunit 5 is sufficient to cause the cop/det/fus mutant phenotype in Arabidopsis. The Plant Cell 17, 1967–1978. 15923347 10.1105/tpc.105.032870 PMC1167545

[CIT0012] Earley KW, Haag JR, Pontes O, Opper K, Juehne T, Song K, Pikaard CS 2006 Gateway-compatible vectors for plant functional genomics and proteomics. The Plant Journal 45, 616–629. 16441352 10.1111/j.1365-313X.2005.02617.x

[CIT0013] Felix G, Boller T 1995 Systemin induces rapid ion fluxes and ethylene biosynthesis in *Lycopersicon peruvianum* cells. The Plant Journal 7, 381–389.

[CIT0014] Gusmaroli G, Feng S, Deng XW 2004 The Arabidopsis CSN5A and CSN5B subunits are present in distinct COP9 signalosome complexes, and mutations in their JAMM domains exhibit differential dominant negative effects on development. The Plant Cell 16, 2984–3001. 15486099 10.1105/tpc.104.025999 PMC527193

[CIT0015] Gusmaroli G, Figueroa P, Serino G, Deng XW 2007 Role of the MPN subunits in COP9 signalosome assembly and activity, and their regulatory interaction with Arabidopsis Cullin3-based E3 ligases. The Plant Cell 19, 564–581. 17307927 10.1105/tpc.106.047571 PMC1867349

[CIT0016] Hamel LP, Nicole MC, Sritubtim S, et al 2006 Ancient signals: comparative genomics of plant MAPK and MAPKK gene families. Trends in Plant Science 11, 192–198. 16537113 10.1016/j.tplants.2006.02.007

[CIT0017] Hann CT, Bequette CJ, Dombrowski JE, Stratmann JW 2014 Methanol and ethanol modulate responses to danger- and microbe-associated molecular patterns. Frontiers in Plant Science 5, 550. 25360141 10.3389/fpls.2014.00550 PMC4197774

[CIT0018] Hettenhausen C, Schuman MC, Wu J 2015 MAPK signaling: a key element in plant defense response to insects. Insect Science 22, 157–164. 24753304 10.1111/1744-7917.12128 PMC5295641

[CIT0019] Hind SR, Pulliam SE, Veronese P, Shantharaj D, Nazir A, Jacobs NS, Stratmann JW 2011 The COP9 signalosome controls jasmonic acid synthesis and plant responses to herbivory and pathogens. The Plant Journal 65, 480–491. 21265900 10.1111/j.1365-313X.2010.04437.x

[CIT0020] Hoehenwarter W, Thomas M, Nukarinen E, Egelhofer V, Röhrig H, Weckwerth W, Conrath U, Beckers GJ 2013 Identification of novel in vivo MAP kinase substrates in *Arabidopsis thaliana* through use of tandem metal oxide affinity chromatography. Molecular and Cellular Proteomics 12, 369–380. 23172892 10.1074/mcp.M112.020560 PMC3567860

[CIT0021] Holley SR, Yalamanchili RD, Moura DS, Ryan CA, Stratmann JW 2003 Convergence of signaling pathways induced by systemin, oligosaccharide elicitors, and ultraviolet-B radiation at the level of mitogen-activated protein kinases in *Lycopersicon peruvianum* suspension-cultured cells. Plant Physiology 132, 1728–1738. 12913131 10.1104/pp.103.024414 PMC181261

[CIT0022] Ichimura K, Mizoguchi T, Yoshida R, Yuasa T, Shinozaki K 2000 Various abiotic stresses rapidly activate Arabidopsis MAP kinases ATMPK4 and ATMPK6. The Plant Journal 24, 655–665. 11123804 10.1046/j.1365-313x.2000.00913.x

[CIT0023] Kandoth PK, Ranf S, Pancholi SS, Jayanty S, Walla MD, Miller W, Howe GA, Lincoln DE, Stratmann JW 2007 Tomato MAPKs LeMPK1, LeMPK2, and LeMPK3 function in the systemin-mediated defense response against herbivorous insects. Proceedings of the National Academy of Sciences, USA 104, 12205–12210. 10.1073/pnas.0700344104 PMC1924534 17623784

[CIT0024] Kolch W 2005 Coordinating ERK/MAPK signalling through scaffolds and inhibitors. Nature Reviews. Molecular Cell Biology 6, 827–837. 16227978 10.1038/nrm1743

[CIT0025] Kong F, Wang J, Cheng L, Liu S, Wu J, Peng Z, Lu G 2012 Genome-wide analysis of the mitogen-activated protein kinase gene family in *Solanum lycopersicum*. Gene 499, 108–120. 22306326 10.1016/j.gene.2012.01.048

[CIT0026] Lafleur E, Kapfer C, Joly V, Liu Y, Tebbji F, Daigle C, Gray-Mitsumune M, Cappadocia M, Nantel A, Matton DP 2015 The FRK1 mitogen-activated protein kinase kinase kinase (MAPKKK) from *Solanum chacoense* is involved in embryo sac and pollen development. Journal of Experimental Botany 66, 1833–1843. 25576576 10.1093/jxb/eru524 PMC4378624

[CIT0027] Lampard GR, Macalister CA, Bergmann DC 2008 Arabidopsis stomatal initiation is controlled by MAPK-mediated regulation of the bHLH SPEECHLESS. Science 322, 1113–1116. 19008449 10.1126/science.1162263

[CIT0028] Lassowskat I, Böttcher C, Eschen-Lippold L, Scheel D, Lee J 2014 Sustained mitogen-activated protein kinase activation reprograms defense metabolism and phosphoprotein profile in *Arabidopsis thaliana*. Frontiers in Plant Science 5, 554. 25368622 10.3389/fpls.2014.00554 PMC4202796

[CIT0029] Letourneux C, Rocher G, Porteu F 2006 B56-containing PP2A dephosphorylate ERK and their activity is controlled by the early gene IEX-1 and ERK. The EMBO Journal 25, 727–738. 16456541 10.1038/sj.emboj.7600980 PMC1383561

[CIT0030] Leung J, Orfanidi S, Chefdor F, Meszaros T, Bolte S, Mizoguchi T, Shinozaki K, Giraudat J, Bogre L 2006 Antagonistic interaction between MAP kinase and protein phosphatase 2C in stress recovery. Plant Science 171, 596–606.

[CIT0031] Liu Y, Schiff M, Dinesh-Kumar SP 2002 Virus-induced gene silencing in tomato. The Plant Journal 31, 777–786. 12220268 10.1046/j.1365-313x.2002.01394.x

[CIT0032] Lumbreras V, Vilela B, Irar S, Solé M, Capellades M, Valls M, Coca M, Pagès M 2010 MAPK phosphatase MKP2 mediates disease responses in Arabidopsis and functionally interacts with MPK3 and MPK6. The Plant Journal 63, 1017–1030. 20626661 10.1111/j.1365-313X.2010.04297.x

[CIT0033] **MAPK Group** 2002 Mitogen-activated protein kinase cascades in plants: a new nomenclature. Trends in Plant Science 7, 301–308. 12119167 10.1016/s1360-1385(02)02302-6

[CIT0034] Meng X, Shan L, He P 2015 Stack heterotrimeric G proteins and MAPK cascades on a RACK. Molecular Plant 8, 1691–1693. 26612253 10.1016/j.molp.2015.11.005 PMC5156934

[CIT0035] Meng X, Zhang S 2013 MAPK cascades in plant disease resistance signaling. Annual Review of Phytopathology 51, 245–266. 10.1146/annurev-phyto-082712-102314 23663002

[CIT0036] Nakagami H, Kiegerl S, Hirt H 2004 OMTK1, a novel MAPKKK, channels oxidative stress signaling through direct MAPK interaction. Journal of Biological Chemistry 279, 26959–26966. 15033984 10.1074/jbc.M312662200

[CIT0037] Nguyen A, Burack WR, Stock JL, et al 2002 Kinase suppressor of Ras (KSR) is a scaffold which facilitates mitogen-activated protein kinase activation in vivo. Molecular and Cellular Biology 22, 3035–3045. 11940661 10.1128/MCB.22.9.3035-3045.2002 PMC133772

[CIT0038] Oh CS, Pedley KF, Martin GB 2010 Tomato 14-3-3 protein 7 positively regulates immunity-associated programmed cell death by enhancing protein abundance and signaling ability of MAPKKK α. The Plant Cell 22, 260–272. 20061552 10.1105/tpc.109.070664 PMC2828692

[CIT0039] Pedley KF, Martin GB 2004 Identification of MAPKs and their possible MAPK kinase activators involved in the Pto-mediated defense response of tomato. Journal of Biological Chemistry 279, 49229–49235. 15371431 10.1074/jbc.M410323200

[CIT0040] Pereira SR, Vasconcelos VM, Antunes A 2011 The phosphoprotein phosphatase family of Ser/Thr phosphatases as principal targets of naturally occurring toxins. Critical Reviews in Toxicology 41, 83–110. 21288162 10.3109/10408444.2010.515564

[CIT0041] Popescu SC, Popescu GV, Bachan S, Zhang Z, Gerstein M, Snyder M, Dinesh-Kumar SP 2009 MAPK target networks in *Arabidopsis thaliana* revealed using functional protein microarrays. Genes and Development 23, 80–92. 19095804 10.1101/gad.1740009 PMC2632172

[CIT0042] Posas F, Saito H 1997 Osmotic activation of the HOG MAPK pathway via Ste11p MAPKKK: scaffold role of Pbs2p MAPKK. Science 276, 1702–1705. 9180081 10.1126/science.276.5319.1702

[CIT0043] Qiu JL, Fiil BK, Petersen K, et al 2008 Arabidopsis MAP kinase 4 regulates gene expression through transcription factor release in the nucleus. EMBO Journal 27, 2214–2221. 18650934 10.1038/emboj.2008.147 PMC2519101

[CIT0044] Rao KP, Richa T, Kumar K, Raghuram B, Sinha AK 2010 In silico analysis reveals 75 members of mitogen-activated protein kinase kinase kinase gene family in rice. DNA Research 17, 139–153. 20395279 10.1093/dnares/dsq011 PMC2885274

[CIT0045] Ren D, Yang KY, Li GJ, Liu Y, Zhang S 2006 Activation of Ntf4, a tobacco mitogen-activated protein kinase, during plant defense response and its involvement in hypersensitive response-like cell death. Plant Physiology 141, 1482–1493. 16798947 10.1104/pp.106.080697 PMC1533962

[CIT0046] Robbins DJ, Zhen E, Owaki H, Vanderbilt CA, Ebert D, Geppert TD, Cobb MH 1993 Regulation and properties of extracellular signal-regulated protein kinases 1 and 2 in vitro. Journal of Biological Chemistry 268, 5097–5106. 8444886

[CIT0047] Rodriguez MC, Petersen M, Mundy J 2010 Mitogen-activated protein kinase signaling in plants. Annual Review of Plant Biology 61, 621–649. 10.1146/annurev-arplant-042809-112252 20441529

[CIT0048] Schweighofer A, Kazanaviciute V, Scheikl E, et al 2007 The PP2C-type phosphatase AP2C1, which negatively regulates MPK4 and MPK6, modulates innate immunity, jasmonic acid, and ethylene levels in Arabidopsis. The Plant Cell 19, 2213–2224. 17630279 10.1105/tpc.106.049585 PMC1955703

[CIT0049] Shaw AS, Filbert EL 2009 Scaffold proteins and immune-cell signalling. Nature Reviews. Immunology 9, 47–56. 10.1038/nri2473 19104498

[CIT0050] Sinha AK, Jaggi M, Raghuram B, Tuteja N 2011 Mitogen-activated protein kinase signaling in plants under abiotic stress. Plant Signaling and Behavior 6, 196–203. 21512321 10.4161/psb.6.2.14701 PMC3121978

[CIT0051] Stanko V, Giuliani C, Retzer K, Djamei A, Wahl V, Wurzinger B, Wilson C, Heberle-Bors E, Teige M, Kragler F 2014 Timing is everything: highly specific and transient expression of a MAP kinase determines auxin-induced leaf venation patterns in Arabidopsis. Molecular Plant 7, 1637–1652. 25064848 10.1093/mp/ssu080 PMC4228985

[CIT0052] Stratmann J 2008 MAP kinases in plant responses to herbivory. In: Schaller A, ed. Induced plant resistance to herbivory. Berlin: Springer, 329–347.

[CIT0053] Stratmann JW, Ryan CA 1997 Myelin basic protein kinase activity in tomato leaves is induced systemically by wounding and increases in response to systemin and oligosaccharide elicitors. Proceedings of the National Academy of Sciences, USA 94, 11085–11089. 10.1073/pnas.94.20.11085 PMC23618 9380763

[CIT0054] Stulemeijer IJ, Stratmann JW, Joosten MH 2007 Tomato mitogen-activated protein kinases LeMPK1, LeMPK2, and LeMPK3 are activated during the Cf-4/Avr4-induced hypersensitive response and have distinct phosphorylation specificities. Plant Physiology 144, 1481–1494. 17478632 10.1104/pp.107.101063 PMC1914120

[CIT0055] Su J, Xu J, Zhang S 2015 RACK1, scaffolding a heterotrimeric G protein and a MAPK cascade. Trends in Plant Science 20, 405–407. 25986967 10.1016/j.tplants.2015.05.002

[CIT0056] Umbrasaite J, Schweighofer A, Kazanaviciute V, et al 2010 MAPK phosphatase AP2C3 induces ectopic proliferation of epidermal cells leading to stomata development in Arabidopsis. PLoS One 5, e15357. 21203456 10.1371/journal.pone.0015357 PMC3009721

[CIT0057] Wang H, Liu Y, Bruffett K, Lee J, Hause G, Walker JC, Zhang S 2008 Haplo-insufficiency of MPK3 in MPK6 mutant background uncovers a novel function of these two MAPKs in Arabidopsis ovule development. The Plant Cell 20, 602–613. 18364464 10.1105/tpc.108.058032 PMC2329925

[CIT0058] Wang H, Ngwenyama N, Liu Y, Walker JC, Zhang S 2007 Stomatal development and patterning are regulated by environmentally responsive mitogen-activated protein kinases in Arabidopsis. The Plant Cell 19, 63–73. 17259259 10.1105/tpc.106.048298 PMC1820971

[CIT0059] Whitmarsh AJ 2006 The JIP family of MAPK scaffold proteins. Biochemical Society Transactions 34, 828–832. 17052208 10.1042/BST0340828

[CIT0060] Willoughby EA, Perkins GR, Collins MK, Whitmarsh AJ 2003 The JNK-interacting protein-1 scaffold protein targets MAPK phosphatase-7 to dephosphorylate JNK. Journal of Biological Chemistry 278, 10731–10736. 12524447 10.1074/jbc.M207324200

[CIT0061] Wu J, Wang J, Pan C, Guan X, Wang Y, Liu S, He Y, Chen J, Chen L, Lu G 2014 Genome-wide identification of MAPKK and MAPKKK gene families in tomato and transcriptional profiling analysis during development and stress response. PLoS One 9, e103032. 25036993 10.1371/journal.pone.0103032 PMC4103895

[CIT0062] Xu J, Zhang S 2015 Mitogen-activated protein kinase cascades in signaling plant growth and development. Trends in Plant Science 20, 56–64. 25457109 10.1016/j.tplants.2014.10.001

[CIT0063] Yalamanchili RD, Stratmann JW 2002 Ultraviolet-B activates components of the systemin signaling pathway in *Lycopersicon peruvianum* suspension-cultured cells. Journal of Biological Chemistry 277, 28424–28430. 12034744 10.1074/jbc.M203844200

[CIT0064] Zeke A, Lukács M, Lim WA, Reményi A 2009 Scaffolds: interaction platforms for cellular signalling circuits. Trends in Cell Biology 19, 364–374. 19651513 10.1016/j.tcb.2009.05.007 PMC3073007

[CIT0065] Zhou C, Cai Z, Guo Y, Gan S 2009 An arabidopsis mitogen-activated protein kinase cascade, MKK9–MPK6, plays a role in leaf senescence. Plant Physiology 150, 167–177. 19251906 10.1104/pp.108.133439 PMC2675715

