# Supplementary material for: MAP kinases associate with high molecular weight multiprotein complexes
Source: J Exp Bot. 2017 Dec 12;69(3):643–54. doi: 10.1093/jxb/erx424 (PMC5853780; doi:10.1093/jxb/erx424)

Supplementary Figure Legends

Supplementary Fig. S1. Phylogenetic analysis of AtMPK6, 10, and orthologs. (A) Amino acid alignment of the C-termini of various MAPKs. Identical and conserved amino acids are shaded in black and gray, respectively. Numbers on the right side show length of complete amino acid sequence. Dashes represent gaps. The red box outlines the peptide sequence used to generate the anti-AtMPK6 antibody (Sigma-Aldrich). MPK6 and MPK10 share eight amino acids within this sequence. (B) Phylogenetic tree of MAPK proteins. Branches are annotated with bootstrap support (1000 replicates). Prefixes: At - *Arabidopsis thaliana*; Sl -*Solanum lycopersicum;* Nt - *Nicotiana tabacum*.

Supplementary Fig. S2. Reduction of monomeric and complex-associated SlMPK2 in *VIGS-mpk1/2/3* plants. GF/IB analysis of leaf extracts (1.5 mg total protein) from tomato plants in which *MPK1*, *MPK2*, and *MPK3* were co-silenced via VIGS (*VIGS-mpk1/2/3)* and from control plants. SlMPK1/2 were detected using anti-SlMPK2 antibody, which specifically detects SlMPK2 but not SlMPK1 (Holley *et al.*, 2003)**.** Note that the Coomassie-stained (CBB) proteins reflect the elution profile from the GF column with the prominent Rubisco band eluting in HMW GF fractions. The presence of large amounts of Rubisco is a possible cause for the MAPK band depression in HMW fractions. Similar results were obtained in 2 independent experiments.

Supplementary Fig. S3. MAP kinase-containing multiprotein complexes in *N. benthamiana*. (A,B) GF/IB analysis of leaf extracts (1 mg total protein) from *N. benthamiana*. Total protein was extracted from leaf tissue and separated by GF. Eluate was collected in 0.5 mL fractions, concentrated, and analyzed by immunoblotting (IB). Only fractions representing 13 or 13.5 to 18 mL are shown (numbers underneath IBs). The numbers above the panels indicate the peak elution of MW standards in kDa. Input samples represent 30 μg of total protein extracted from leaf tissue and represent the same protein extracts that were used for GF. (A) SIPK/Ntf4 were detected using anti-AtMPK6 antibody. Asterisk * indicates IB lane of unrelated experiment. Note: These plants were transiently transformed with a tomato MAPK-GFP fusion construct, which was poorly

expressed and eluated at higher MW. The bands shown represent the *N. benthamiana* wild type SIPK/Ntf4 bands. (B) FLAG-tagged SlMPK1 protein associates with a multiprotein complex. GF/IB analysis of extracts from WT and from *35S:SlMPK1-FLAG* (MPK1-FLAG) overexpressing leaves, probed with anti-FLAG antibody. *35S:SlMPK1-FLAG* fusions were introduced via *Agrobacterium* and transiently expressed in *N. benthamiana.* Protein was extracted from non-infiltrated *N. benthamiana* leaves (WT) or leaves overexpressing MPK fusion proteins. (C) Immunoprecipitation of FLAG-tagged proteins from pooled HMW (13-14.5 mL) or LMW (15.5-17 mL) GF fractions with anti-FLAG antibody-conjugated beads, followed by IB with anti-FLAG antibody. * indicates IgG band. Results shown in (B) were repeated in two independent experiments with similar results, results shown in (C) were not repeated.

Supplementary Fig. S4. Characterization of Arabidopsis T-DNA insertion lines *mpk10-1* and *mpk10-2*. (A) Gene structure of *MPK10* T-DNA insertion SALK lines *mpk10-1 and mpk10-2*. T-DNA insertions are shown by large triangles. Exons are shown as black boxes and introns as lines between exons. In *mpk10-1,* the T-DNA inserted in the first exon. In *mpk10-2*, the T-DNA inserted in the promoter region. (B) T-DNA insertion and homozygosity of *mpk10* mutants. PCR reactions were carried out to amplify *MPK10* sequences from Col-0, *mpk10-1*, and *mpk10-2* genomic DNA using specific left and right primers (LP and RP). T-DNA inserts were detected by PCR on *mpk10* mutants using primers LBb1.3 and RP. Mutants were determined to be homozygous due to amplification of the T-DNA insert in *mpk10-1* (0.8 kb band) and *mpk10-2* (0.6 kb band) and the lack of MPK10 amplification from *mpk10-1* (no band at 1.1 kb using LP+RP+LBb1.3 primers) and *mpk10-2* (no band at 1.2 kb using LP+RP primers). Note: PCR amplification of *MPK10* and the T-DNA insert from *mpk10-2* required separate reactions of LP+RP and RP+LBb1.3 for optimal results. Approximate band sizes were determined using a 1kb DNA ladder for reference.

Supplementary Fig. S5. GF/IB analyses showing that unphosphorylated MPKs associate with a multiprotein MAPK complex in response to UV-C, wounding, and systemin. (A) GF/IB analysis of extracts (1.4 mg total protein) from untreated (Unt, 0) *S. peruvianum*

suspension-cultured cells or cells irradiated for 5 minutes with UV-C radiation and extracted 60 min after onset of UV-C irradiation probed with anti-pERK antibody for the detection of phosphorylated MPK1/2 (p-MPK1/2). Inputs represent 30 μg of total protein extracted from cells collected at the times indicated after treatment. Numbers above and below IBs are as described in **Fig. 1** and **Fig. S3**. (B) GF/IB analysis of extracts (1.2 mg total protein) from tobacco leaves sampled 180 min after leaf wounding (Wdg), probed with anti-AtMPK6 antibody for the detection of SIPK/Ntf4 or anti-pERK antibody for the detection of phosphorylated SIPK/Ntf4 (p-SIPK/Ntf4). Inputs show samples at 0, 10, and 180 min after wounding. Input bands probed with anti-AtMPK6 became visible after longer exposure time. GF/IB analyses for 0 and 10 min are shown in Fig. 4C. * Unrelated protein that cross-reacts with anti-AtMPK6 antibodies. (C) Enzymatic activity of MPK1/2 in extracts (1.5 mg total protein) from tomato leaves as determined by GF followed by an in-gel kinase assays (IGKA). Leaves were left untreated (Unt) or wounded (Wdg) and sampled 10 min later. Total protein (1.5 mg) was separated by GF and each fraction was analyzed by IGKA. Signals represent ^{32}P-phosphorylated myelin basic protein. (D) GF/IB analysis of extracts (0.75 mg total protein) from *S. peruvianum* suspension-cultured cells, probed with anti-AtMPK6 antibody. Cells were treated with 10 nM systemin. Total protein was extracted from suspension cells 180 min after treatment. Inputs represent 30 μg of total protein extracted at 180 min after systemin treatment. Numbers above and below IBs are as described in Fig. 1.

Supplementary Figure S1

A.

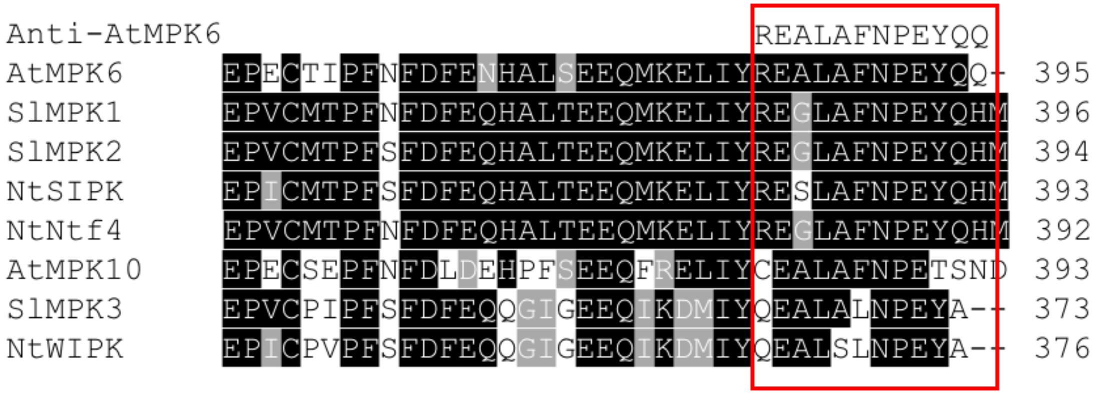

B.

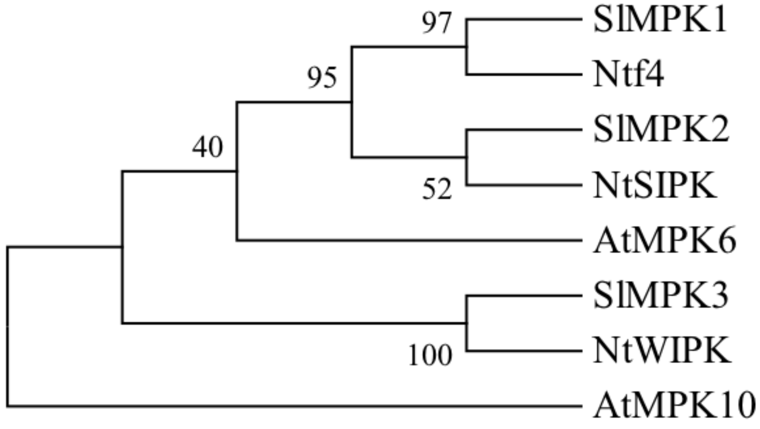

Supplementary Fig. S2

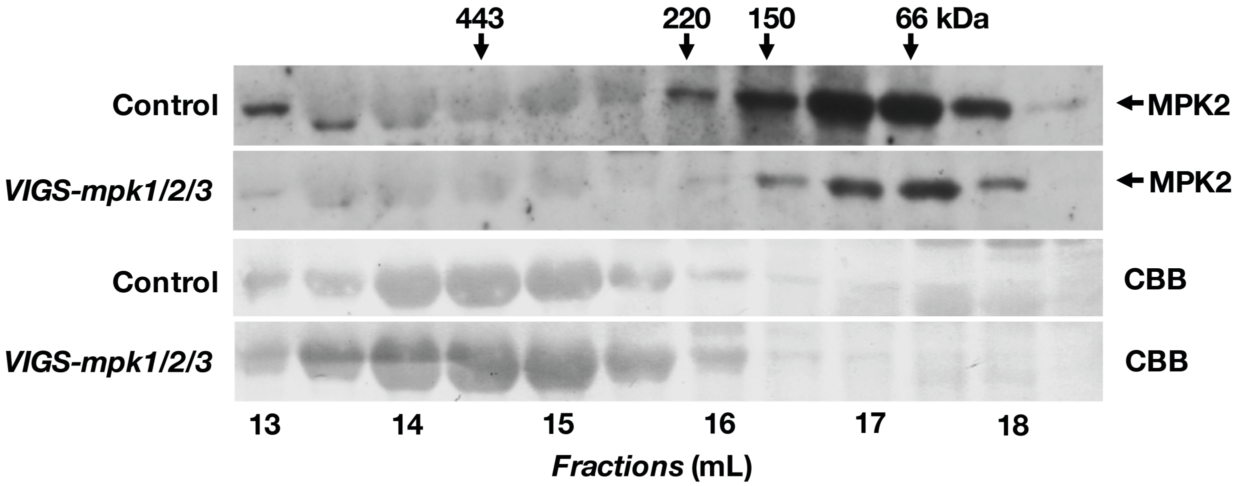

Supplementary Fig. S3

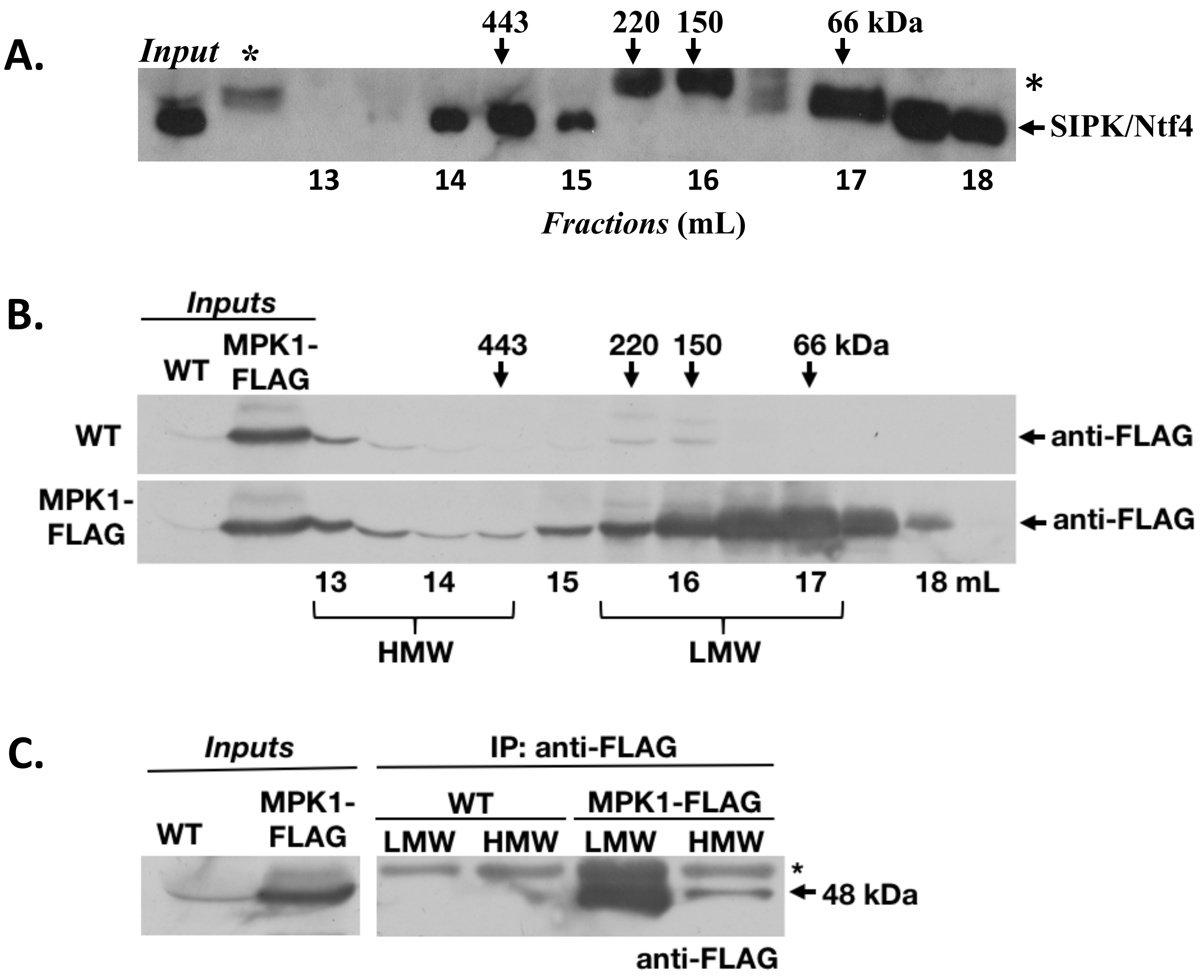

Supplementary Fig. S4

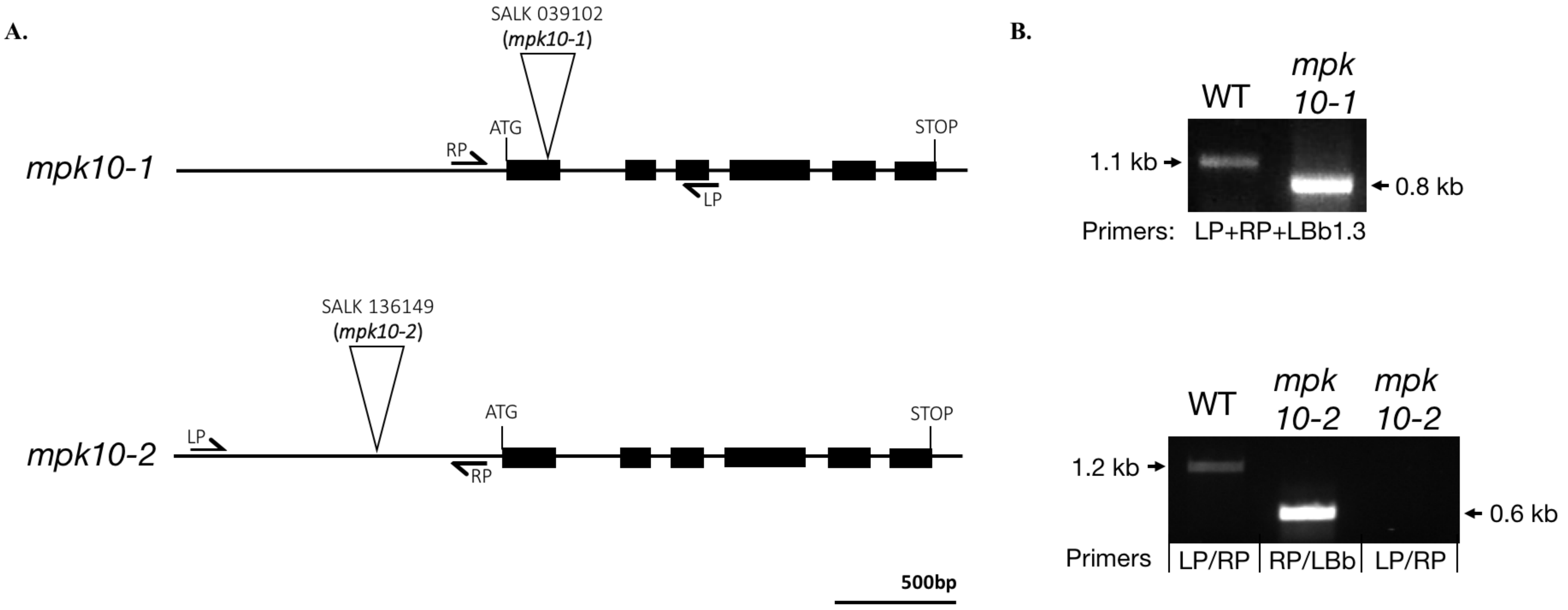

Supplementary Fig. S5

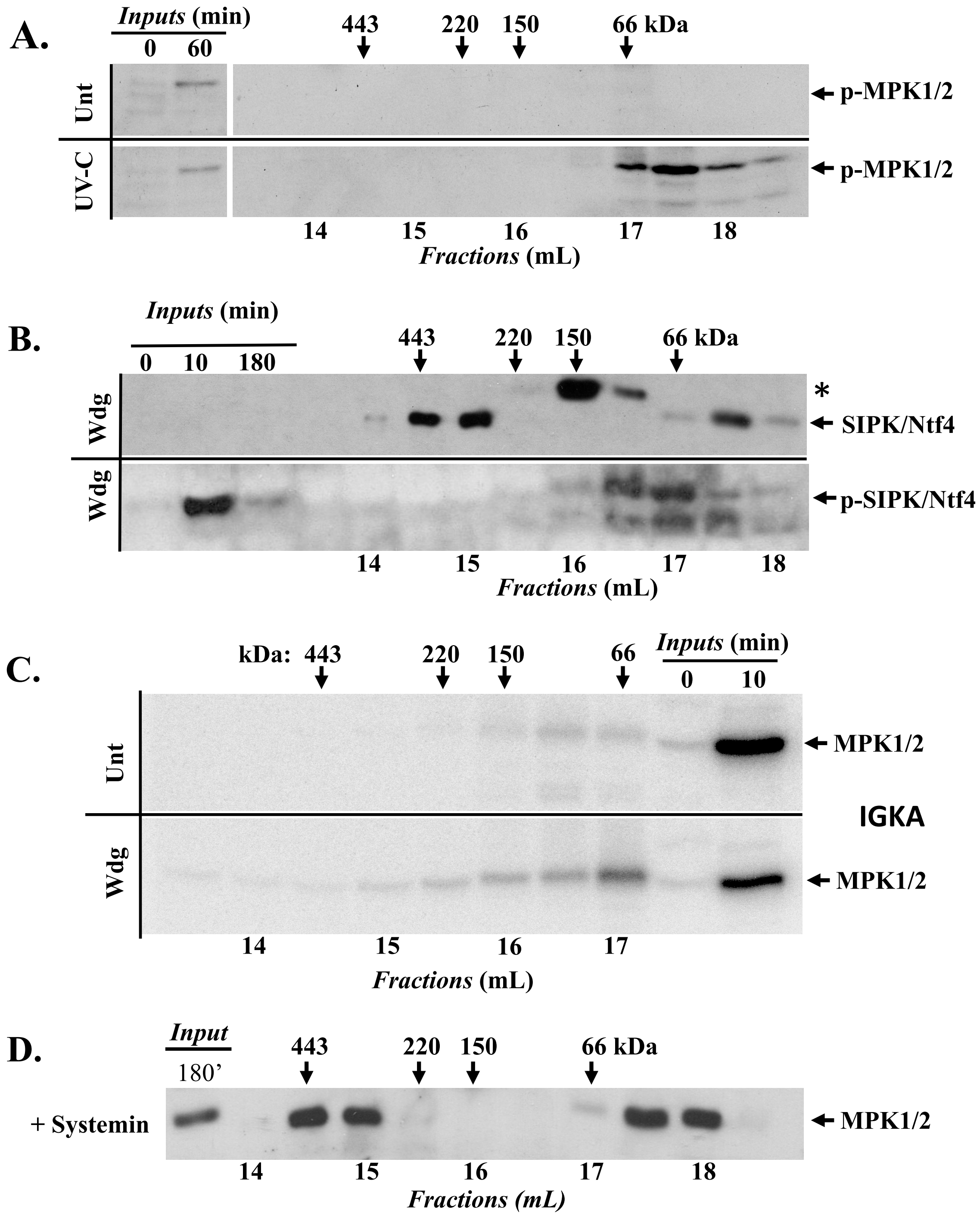

Supplement: supplementary-figures-S1-S5 [file erx424_suppl_supplementary-figures-s1-s5.pdf]
